# Genome-wide analyses of behavioural traits are subject to bias by misreports and longitudinal changes

**DOI:** 10.1038/s41467-020-20237-6

**Published:** 2021-01-07

**Authors:** Angli Xue, Longda Jiang, Zhihong Zhu, Naomi R. Wray, Peter M. Visscher, Jian Zeng, Jian Yang

**Affiliations:** 1 Institute for Molecular Bioscience, The University of Queensland, Brisbane, QLD 4072 Australia; 2 Queensland Brain Institute, The University of Queensland, Brisbane, QLD 4072 Australia; 3 School of Life Sciences, Westlake University, Hangzhou, Zhejiang 310024 China; 4 Westlake Laboratory of Life Sciences and Biomedicine, Hangzhou, Zhejiang 310024 China

**Keywords:** Genetic association study, Population genetics, Quantitative trait, Risk factors

## Abstract

Genome-wide association studies (GWAS) have discovered numerous genetic variants associated with human behavioural traits. However, behavioural traits are subject to misreports and longitudinal changes (MLC) which can cause biases in GWAS and follow-up analyses. Here, we demonstrate that individuals with higher disease burden in the UK Biobank (*n* = 455,607) are more likely to misreport or reduce their alcohol consumption levels, and propose a correction procedure to mitigate the MLC-induced biases. The alcohol consumption GWAS signals removed by the MLC corrections are enriched in metabolic/cardiovascular traits. Almost all the previously reported negative estimates of genetic correlations between alcohol consumption and common diseases become positive/non-significant after the MLC corrections. We also observe MLC biases for smoking and physical activities in the UK Biobank. Our findings provide a plausible explanation of the controversy about the effects of alcohol consumption on health outcomes and a caution for future analyses of self-reported behavioural traits in biobank data.

## Abstract (web summary)

Conflicting reports have found disease to sometimes be positively and sometimes negatively correlated with alcohol consumption. Here, the authors show that misreporting and reduction of alcohol consumption is associated with disease, leading to misleading associations between alcohol and disease.

## Introduction

Behaviours and lifestyles are modifiable risk/protective factors for common diseases in humans. In the past few decades, one of the most controversial debates in public health is on the effect of alcohol consumption (AC) on common diseases, especially cardiovascular/metabolic diseases. Large-scale meta-analyses of epidemiological studies^1,2^ on AC concluded that “no level of alcohol consumption improves health”^3^. This conclusion, however, is contradictory to the negative estimates of the genetic correlation (*r*_*g*_) between AC and several diseases such as obesity^4–7^, major depressive disorder (MDD)^6,7^, Parkinson’s disease^5^, and type 2 diabetes (T2D)^5^ reported in the recent genome-wide association studies (GWAS) and also contradictory to the protective effects of moderate drinking reported in observational studies^8,9^. Different hypotheses have been proposed to explain these discrepancies, including (1) heavy AC might alter metabolism or impair nutrient absorption^10,11^, meaning that the effect is dosage-dependent; (2) people who have health problems may quit or reduce drinking, or underreport their intake level^12^; and (3) some other common explanations include confounding factors^6^ (e.g., socioeconomic status and physical activities) and collider bias^13,14^. Nevertheless, to date, no study has provided an in-depth investigation into the causes of the discrepancies.

In epidemiological or genetic studies, phenotypic data of behavioural and lifestyle traits are often collected from self-reported questionnaires, which are subject to misreports (i.e., self-report biases), especially for questions related to smoking, drinking, and drug use^15–18^. These phenotypes are also subject to change during lifetime^19–22^, for instance in response to disease diagnosis, but data to track such longitudinal variations are rarely available. Both misreports and longitudinal changes (hereafter referred to as MLC) could change the distribution of the phenotypes and thus may affect the results of both epidemiological and genetic studies.

In this study, we set out to investigate biases due to MLC in genetic analyses of self-reported behavioural traits including AC, tobacco smoking, and physical activities in the UK Biobank (UKB)^23^. The UKB includes detailed questionnaires of these behavioural traits, providing a unique resource to investigate the potential pitfalls in the analyses of self-reported phenotypes. We demonstrate that MLC could induce biases in GWAS of these traits and follow-up analyses that use summary statistics from the GWAS. We then propose a correction procedure to mitigate the MLC biases. Using AC as an example, we identify and remove the participants whose self-reported AC is inconsistent with their intake frequency, medical records or online follow-ups and the participants who reduced their AC intake because of illness or doctor’s advice during the past 10 years. Then, we stratify the participants into three longitudinal change groups (drink “less”, “the same” or “more” compared to 10 years ago) and run a GWAS analysis in each group separately followed by a meta-analysis. We also elaborate on why some of the previous studies might suffer from MLC biases.

## Results

### Misreports and longitudinal changes in alcohol consumption

Misreports are common in self-reported data sets^15–18^ but often overlooked in genetic analyses. Here, we focused on the analyses of AC because (1) its relationship with common diseases is controversial; (2) the data required by our investigations and corrections are available; (3) the sample size is large (*n* = 455,607). In this study, our definition of misreports for AC includes misreporting about drinking status^24^, underreporting the AC level^15,17^, and selective recall of the question about AC level^25^, all of which might occur in the UKB. These kinds of misreports are mainly attributed to^26,27^ social desirability^28,29^ (i.e., the tendency of participants to answer questions in ways that make them viewed favourably by others) and recall bias^30,31^ (i.e., the accuracy and completeness of past events recalled by participants are influenced by subsequent events that they experienced). First, 14,488 UKB participants identified themselves as never drinkers, but data from follow-up questionnaires and medical records^32^ for 3,627 of these participants suggested that at least 10% of the individuals were very likely to have drinking history, e.g., previously diagnosed as having alcoholic hepatitis or alcohol use disorder (Supplementary Note 1). This result validated a previous conclusion that classifying self-reported never drinkers as lifetime abstainers could be problematic^24^. Thus, our analyses of AC were mainly focused on current drinkers (*n* = 424,507) unless specified elsewhere. Second, 9,064 individuals (2.1%) were classified as current drinkers but reported zero consumption level, indicating possible underreporting. Third, 66,058 individuals (15.6%) reported their alcohol intake frequency and other related questions but did not report their actual AC levels, suggesting a potential selective recall bias. It has been shown previously that heavy drinkers tend to be less responsive^25^, and a high non-response rate could lead to an underestimate of the average AC level in the sample^33^. To investigate the characteristics of the suspected misreporting individuals, we examined the phenotypes of 18 common diseases in the UKB and used disease count (the number of diseases carried) as an indicator of disease burden for each participant (Methods; Table 1 and Supplementary Data 1). We observed that unresponsive individuals had a significantly higher mean disease count than individuals with complete responses (1.63 vs. 1.37, Welch *t*-test *P* = 6.35 × 10^−294^; Table 1). The suspected underreporting individuals (*n* = 9,064) also showed a significantly higher mean disease count than the remaining current drinkers (1.73 vs. 1.36, Welch *t*-test *P* = 2.68 × 10^−87^).

**Table 1:** Alcohol consumption and health-related traits of current drinkers in different response and longitudinal change groups.

Intake level reported	Longitudinal change group	*N*	Mean phenotype			Disease prevalence (%)			
			AC	BMI	Disease count	CVD	T2D	DYSLIPID	HYPER
Yes	All	358,449	10.67 (0.02)	27.17 (0.01)	1.372 (0.002)	15.42 (0.06)	5.13 (0.04)	16.91 (0.06)	18.36 (0.06)
	LESS	152,854 (42.6%)	8.56 (0.02)	27.77 (0.01)	1.506 (0.004)	17.80 (0.10)	7.20 (0.07)	19.17 (0.10)	21.12 (0.10)
	SAME	134,808 (37.6%)	10.82 (0.03)	26.72 (0.01)	1.274 (0.004)	14.08 (0.09)	3.83 (0.05)	15.76 (0.10)	16.71 (0.10)
	MORE	68,855 (19.2%)	15.11 (0.05)	26.68 (0.02)	1.266 (0.005)	12.77 (0.13)	3.10 (0.07)	14.12 (0.13)	15.40 (0.14)
No	All	66,058	—	28.28 (0.02)	1.630 (0.007)	17.70 (0.15)	8.16 (0.11)	18.07 (0.15)	21.27 (0.16)
	LESS	38,799 (58.7%)	—	28.67 (0.03)	1.730 (0.009)	19.36 (0.20)	9.60 (0.15)	19.50 (0.20)	22.87 (0.21)
	SAME	25,055 (37.9%)	—	27.70 (0.03)	1.474 (0.010)	15.26 (0.23)	6.09 (0.15)	15.93 (0.23)	18.93 (0.25)
	MORE	1599 (2.4%)	—	27.43 (0.13)	1.448 (0.039)	13.82 (0.86)	4.88 (0.54)	14.70 (0.89)	16.20 (0.92)

Shown are the descriptive characteristics of alcohol consumption and health-related traits. The numbers in the bracket in Mean phenotype and Disease prevalence columns are standard error of each mean/proportion estimate. LESS, SAME, and MORE denote the current drinkers who reduced, maintained, and increased their alcohol consumption, respectively, compared to 10 years ago. *N*, sample size; AC, alcohol consumption measured by units per week; BMI, body mass index (kg/m^2^); Disease count, number of common diseases affected; CVD: cardiovascular disease; T2D: type 2 diabetes; DYSLIPID: dyslipidaemia; and HYPER: hypertensive disease. A tiny proportion (<1%) of participants choose “Do not know” or “Prefer not to answer” are not listed in this Table but can be found in Supplementary Data 1c.

Another important source of bias is the change in drinking volume during the life course for reasons such as changes in health status. For instance, if people change their AC level because they are affected by a disease, such a disease ascertainment will give rise to a bias in observed or genetic relationship between AC and the disease. In the UKB, all the current drinkers (*n* = 424,507) were asked a question “compared to 10 years ago, do you drink less/the same/more nowadays?” (Methods), and 262,107 (61.7%) of them reported “less” or “more”. We denoted the three groups of individuals as LESS, SAME and MORE, respectively. The LESS group (*n* = 191,653, 45.1%) had a lower average AC level, higher disease prevalence for several common diseases, and higher mean disease count than individuals in the other two groups (Table 1 and Supplementary Data 1). A follow-up question was asking the participants to choose the reason(s) why they reduced drinking, and the available options include illness, health precaution, and financial reasons (Table 2). There were 15,889 individuals (8.3%) choosing illness or doctor’s advice as the primary reason for reducing drinking, and their mean disease count was nearly twice that of all other current drinkers (Table 2). In the subgroup of individuals who reported AC and had reduced drinking due to illness or doctor’s advice (*n* = 11,886, 3.3%), the prevalence of cardiovascular disease (CVD) was 0.411, ~2.7 times higher than that in all current drinkers (0.154), providing strong evidence of disease ascertainment of AC (Supplementary Data 1).

**Table 2:** Descriptive statistics of the reasons for reducing alcohol intake.

Intake level reported	Reason	*N*	Mean phenotype			
			AC	BMI	Disease count	EA
Yes	Illness or ill health	8555 (5.6%)	7.33 (0.10)	28.53 (0.06)	2.77 (0.02)	12.81 (0.06)
	Doctor’s advice	3331 (2.2%)	14.72 (0.23)	29.49 (0.09)	2.58 (0.03)	12.65 (0.09)
	Health precaution	48,483 (31.7%)	9.98 (0.04)	27.44 (0.02)	1.54 (0.01)	14.17 (0.02)
	Financial reasons	7323 (4.8%)	10.27 (0.12)	28.54 (0.06)	1.74 (0.02)	11.57 (0.06)
	Other reason	68,066 (44.6%)	7.54 (0.03)	27.71 (0.02)	1.28 (0.01)	13.90 (0.02)
	Prefer not to answer	268 (0.2%)	5.77 (0.45)	27.69 (0.30)	1.75 (0.10)	10.90 (0.30)
	Do not know	16,729 (11.0%)	7.28 (0.06)	27.96 (0.04)	1.35 (0.01)	13.00 (0.04)
No	Illness or ill health	3541 (9.1%)	—	29.28 (0.10)	2.81 (0.03)	12.35 (0.09)
	Doctor’s advice	462 (1.2%)	—	29.99 (0.26)	2.77 (0.09)	11.90 (0.24)
	Health precaution	6767 (17.5%)	—	28.26 (0.06)	1.87 (0.02)	12.86 (0.06)
	Financial reasons	1600 (4.1%)	—	29.05 (0.14)	1.91 (0.05)	11.26 (0.12)
	Other reason	21,362 (55.1%)	—	28.62 (0.04)	1.51 (0.01)	12.88 (0.03)
	Prefer not to answer	101 (0.3%)	—	28.23 (0.53)	2.10 (0.17)	9.56 (0.41)
	Do not know	4934 (12.7%)	—	28.79 (0.08)	1.53 (0.02)	11.89 (0.07)

The proportion in the bracket in *N* column indicates the proportion of each reason within two categories (reported intake level or not). The numbers in the bracket in Mean phenotype column are standard error of each mean estimate. *N*, sample size; AC, alcohol consumption (units per week); BMI, body mass index (kg/m^2^); Disease count, number of common diseases affected; EA, educational attainment (years of schooling). A total of 132 individuals did not have records for their reasons for reducing their alcohol intake.

### Biases in GWAS for alcohol consumption due to MLC

**Fig. 1:**
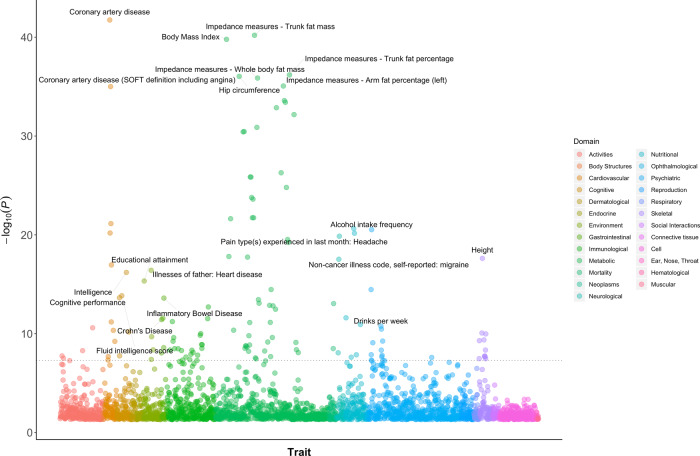
PheWAS results for the 16 AC GWAS signals that became non-significant after the MLC corrections. This figure shows associations of the AC-associated variants, which became non-significant because of the MLC corrections, with all the common traits and diseases for which summary data from large-scale GWASs are available in the public domain (https://atlas.ctglab.nl/PheWAS). The colour denotes the domain of each associated trait. There were 136 traits associated with the 16 SNPs with $P\, <\, 5 \times 10^{ - 8}$, and 61 (44.9%) of them were metabolic/cardiovascular traits.

We conducted GWAS analyses for AC with and without correcting for MLC. The MLC corrections included excluding individuals who might underreport AC level, excluding individuals who reduced drinking due to illness or doctor’s advice, and adjusting the mean and variance difference in the three longitudinal change groups (Methods and Supplementary Figs. 1–2). There were 53 and 47 independently genome-wide significant loci ($P_{{\mathrm{GWAS}}}\, <\, 5 \times 10^{ - 8}$) before and after the corrections, respectively (Supplementary Data 2 and Supplementary Figure 3). We identified 16 loci that became non-significant after the corrections ($P_{{\mathrm{GWAS}}} \ge 5 \times 10^{ - 8}$, Supplementary Data 2). By searching the top associated SNPs at these loci in an online database PheWAS^34^ (http://atlas.ctglab.nl/PheWAS), we found that 44.9% of associated phenotypes ($P_{{\mathrm{PheWAS}}}\, <\, 5 \times 10^{ - 8}$) were metabolic/cardiovascular traits such as body mass index (BMI), triglyceride (TC), and coronary artery disease (CAD) (Fig. 1). We showed by a down-sampling analysis that the number of loci that became non-significant after the MLC corrections (16) was significantly larger than that expected from a loss of sample size (10.03, standard error (s.e.) = 0.49), and 10 loci that became genome-wide significant after the MLC corrections were likely to be masked by MLC in the uncorrected GWAS (the expected number is 3.26, s.e. = 0.30, Methods, Supplementary Data 2–3). These results were in line with the simulation results (Methods and Supplementary Note 2) that MLC could reduce the power to detect true signals and induce spurious signals due to disease ascertainment (Supplementary Figs. 3–9).

### Estimates of genetic correlation biased by MLC

**Fig. 2:**
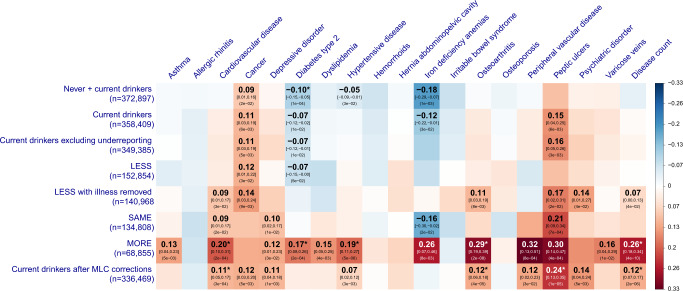
Estimates of genetic correlation between AC and common diseases in the UKB. The rows denote 8 GWAS summary data sets for AC with the sample size labelled in the bracket. The columns are 18 common diseases and disease count. The nominal significant effects ($P\, <\, 0.05$) are labelled with $\hat r_g$ [95% confidence interval] (*P*-value), and the significant effects after multiple testing correction ($P\, <\, 0.05/152$) are labelled with an additional asterisk. The colour of the block represents the size each genetic correlation estimate. The *P*-value shown in the block is the original *P*-value for $\hat r_g$ (two-sided $\chi ^2$ test). “Current drinkers excluding underreporting” represents current drinkers excluding 9,064 individuals who likely underreported their AC levels. LESS, SAME, and MORE represent current drinkers whose AC levels were reduced, maintained the same, and increased, respectively, compared to 10 years ago. “LESS with illness removed” represents the LESS group excluding the participants who reduced their AC intake level due to illness or doctor’s advice.

Biases in GWAS results due to MLC are expected to carry over to follow-up analyses using summary statistics of the GWAS, such as the genetic correlation ($r_g$) analysis. To demonstrate such biases, we estimated $r_g$ between AC and 18 common diseases in the UKB by the bivariate LD score regression^35^ (LDSC) using AC GWAS data from each of the three longitudinal change groups or the whole sample (Methods). Before the MLC corrections, we observed substantial differences between $\hat r_g$ (between AC and diseases) estimated using AC GWAS data from the LESS, SAME and MORE groups (Fig. 2 and Supplementary Data 4). We also estimated the SNP-based heritability ($h_{SNP}^2$) from different AC GWAS data sets and $r_g$ between the data sets (Supplementary Data 5–6, and Supplementary Fig. 10) and found that the $\hat r_g$ between AC in the LESS and MORE groups was significantly different from unity ($\hat r_g = 0.796$, *s.e*. $= 0.074$). All these results suggested that there was heterogeneity among AC data from the three longitudinal change groups. The heterogeneity was also demonstrated in an additional analysis where we estimated $r_g$ between AC (using data from the UKB) and 234 traits (using data from LD-Hub^36^) and found that the $\hat r_g$ using AC GWAS data from the LESS group were substantially different from those using AC GWAS data from the MORE group, with more than half of the $\hat r_g$ (143/234) in the opposite direction between the two groups (Supplementary Data 7 and Supplementary Fig. 11). Notably, after the MLC corrections, the $\hat r_g$ between all pairwise AC GWAS data sets were close to 1 (ranging from 0.91 to 0.99, Supplementary Data 6), demonstrating the effectiveness of the MLC corrections in eliminating/reducing the biases.

**Fig. 3:**
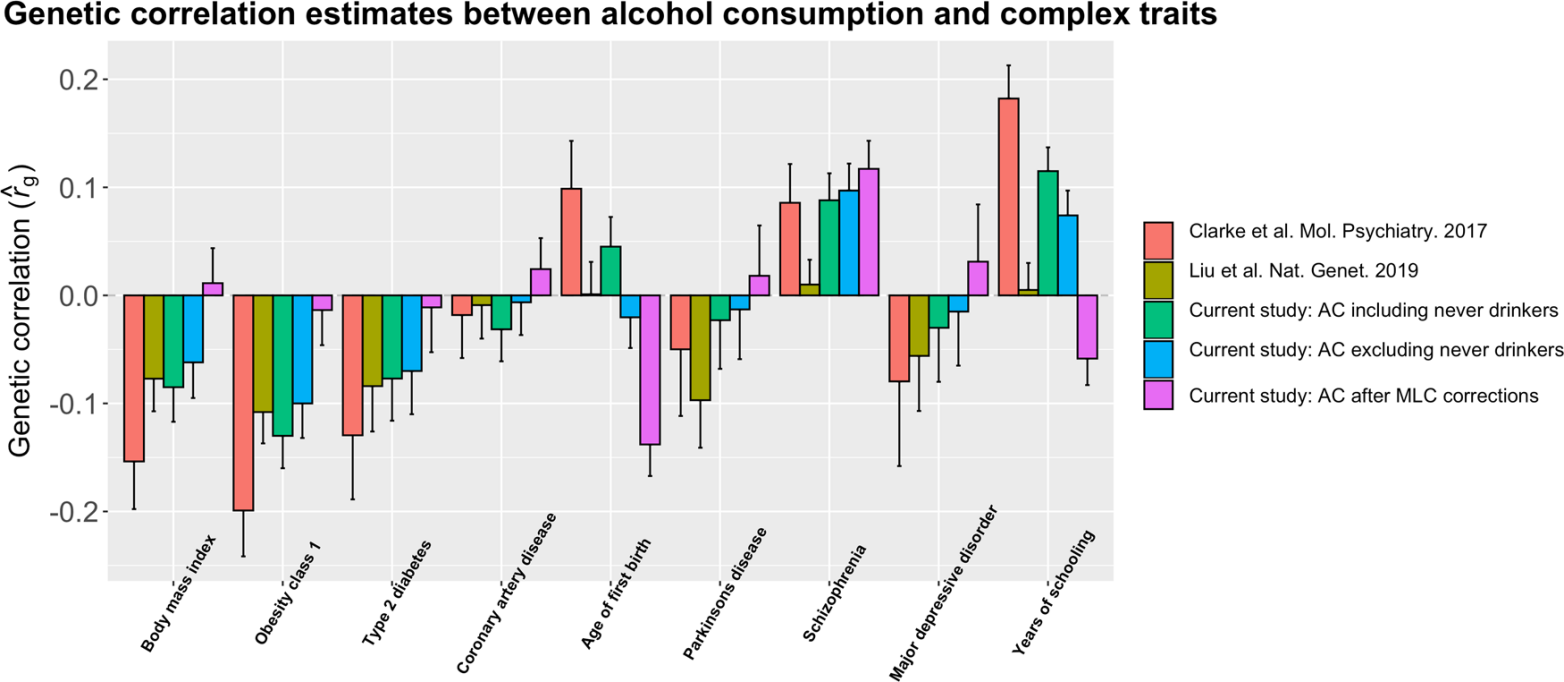
Estimates of genetic correlation between AC and complex traits using data from the UKB and other published studies. Genetic correlation was estimated by the bivariate-LDSC in LD Hub. The y-axis shows the estimate of *r*_g_, and the x-axis shows different complex traits. The error bars denote the standard errors of the estimates. The results using the summary statistics from our analysis were compared to those from Clarke et al.^4^, who used self-reported AC from the interim release of the UKB data, and Liu et al.^5^, a meta-analysis that included the full release of the UKB data. The sample sizes of the five AC data sets are 112,117 (Clarke et al.), 941,280 (Liu et al.), 372,897 (including never drinkers), 358,409 (excluding never drinkers), and 336,469 (after the MLC corrections), respectively.

In the $r_g$ analysis using AC GWAS data from the whole sample without the MLC corrections, AC showed nominally significant (*P* < 0.05) negative $\hat r_g$ with 3 diseases (i.e., T2D, hypertensive disease, and iron deficiency anemias) (Fig. 2 and Supplementary Data 4). However, after the MLC corrections in our study, AC showed nominally significant $\hat r_g$ with 8 diseases as well as disease count, all of which were positive (Fig. 2). Negative estimates of $r_g$ between AC and diseases have also been reported in the literature. For instance, Clarke et al.^4^ showed negative $\hat r_g$ between AC and BMI/obesity, and Liu et al.^5^ showed that AC is negatively genetically correlated with several common diseases including Parkinson’s disease, obesity, and T2D. We show that the negative estimates could be replicated in our study using data without the MLC corrections but most of them turned to positive after the MLC corrections (Fig. 3 and Supplementary Data 8). These results implied that the negative estimates of $r_g$ between AC and diseases from the analyses without the MLC corrections (including those in prior works) were caused by disease ascertainment. Nevertheless, this conclusion was not definitive because the ground truth was unknown in real data analysis. Hence, we sought to verify it by simulation (Methods and Supplementary Note 2), and the results showed that the estimated SNP effect correlation ($\hat r_b$) between a simulated exposure and disease gradually changed to the opposite direction as the strength of disease ascertainment increased (Supplementary Note 2 and Supplementary Figs. 5 and 7), supporting our conclusion. Here $r_b$ is defined as the correlation of true SNP effects between the simulated exposure and disease. The simulation results also showed that after the MLC corrections, $r_b$ was slightly underestimated but with no bias in the direction in the presence of disease ascertainment, and that the MLC corrections did not induce any bias in $\hat r_b$ under the null hypothesis that $r_b = 0$ (Supplementary Fig. 9).

Socioeconomic status (SES) has been shown to affect people’s alcohol use and health outcomes, and several studies have shown that people with higher SES tend to have higher AC levels and lower disease risks than people with lower SES^37,38^. Clarke et al.^4^ and Liu et al.^5^ reported positive $\hat r_g$ between AC and educational attainment (EA). We observed a similar estimate in our study ($\hat r_g = 0.082,\;P = 1.0 \times 10^{ - 4}$) before the MLC corrections, but the estimate became non-significant after the MLC corrections ($\hat r_g = - 0.036,\;P = 0.108,$ Fig. 3), likely because MLC are associated with EA. For example, the mean years of schooling of individuals who reduced AC due to illness/doctor advice (12.76, standard error of the mean $(s.e.m.) = 0.05$) was significantly lower than that of the remaining current drinkers (14.23, $s.e.m. = 0.01$), and people reduced AC because of health precaution had the highest education level than any other reason (Table 2), suggesting that the reasons for reducing intake were EA-dependent. We also included household income (HI) and social deprivation (SD) in the genetic correlation analysis (Methods), and our results showed that $\hat r_g$ between AC and HI or SD were also affected by MLC (Supplementary Data 9).

To test the effects of EA and HI on the $\hat r_g$ between AC and diseases, we adjusted AC for EA and HI. To avoid collider bias due to adjusting for a heritable phenotype, we performed the adjustment using the mtCOJO approach^39^ which is more robust to collider bias than the conventional covariate adjustment approach. We found that before the MLC corrections, the $\hat r_g$ between AC and 18 common diseases after further EA and HI adjustment were highly consistent with those before the adjustment (Pearson’s correlation $r = 0.966$) (Supplementary Fig. 12). The consistency was even higher after the MLC corrections ($r = 0.988$) (Supplementary Fig. 12). These results suggest that biases in AC GWAS due to EA and HI are likely to be small and have largely been removed by the MLC corrections. In addition, there were significant differences in BMI between the LESS and SAME groups (Welch-*t* = 67.9, −log_10_(*P*) = 841.0) and between the LESS and MORE groups (Welch-*t* = 64.0, −log_10_(*P*) = 879.7). The observation is in line with one of our conclusions above that participants with cardiometabolic diseases tend to reduce AC because these diseases are often associated with higher BMI. This observation is unlikely to be driven by EA because the differences remain highly significant (Welch-*t* = 77.3, −log_10_(*P*) = 1288.9 between LESS and SAME and Welch-*t* = 79.5, −log_10_(*P*) = 1344.8 between LESS and MORE) after adjusting BMI for EA.

### Estimates of causal effect biased by MLC

**Fig. 4:**
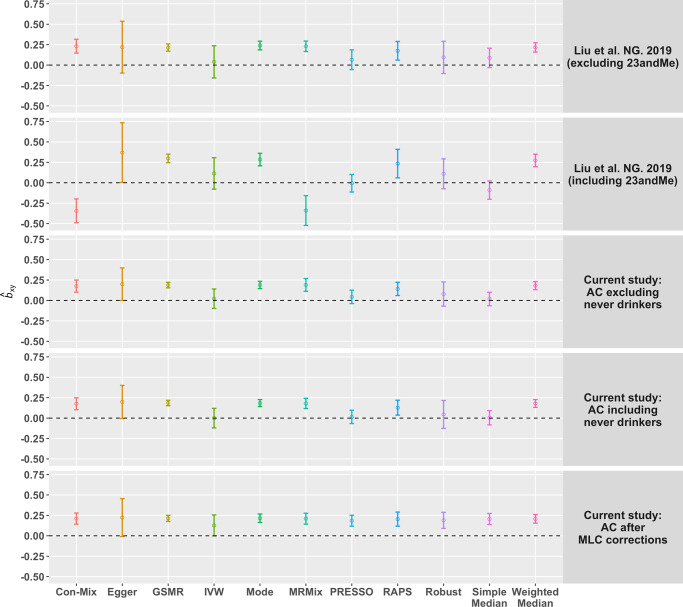
Estimates of causal effect of AC on BMI using different MR methods. The colour of the circle denotes different MR methods. The methods on the x-axis is ranked based on alphabetic order from the left to the right. The y-axis is the *b*_xy_ estimates from each method. The error bars denote 95% confidence interval of the estimates. The row-wise panels indicate five different GWAS summary data sets for AC. The horizontal black dashed line indicates *b*_xy_ = 0. The sample sizes (*n*) of the five AC data sets are 537,349 (Liu et al. excluding 23andMe), 941,280 (Liu et al. including 23andMe), 358,409 (excluding never drinkers), 372,897 (including never drinkers), and 336,469 (after the MLC corrections), respectively.

Mendelian randomisation (MR) is a method that uses genetic variants as instrumental variables (IVs) to infer causal relationship between exposure and outcome^40,41^. As the MR analysis relies on GWAS data, it might also be affected by the MLC biases as described above. We used BMI in the UKB as an example to demonstrate the performance of MR in the presence of MLC, based on several commonly used MR methods including IVW (inverse variance weighted)^42^, Robust^43^, MR-Egger^44^, GSMR^39^, weighted median^45^, simple median^45^, mode^46^, MR-PRESSO^47^, MRMix^48^, Con-Mix^49^, RAPS^50^. While the estimates from some methods (including weighted median, mode and GSMR) were all significantly positive and consistent across all the analyses with or without the MLC corrections, the estimates from IVW, simple median, MR-PRESSO, MRMix, Con-Mix seemed to be sensitive to MLC with some of them being negative (Fig. 4). The negative estimates from the analyses without the MLC corrections were likely to be driven by the 16 loci that were removed by the MLC corrections (note that the mean per-SNP MR estimate for the 16 loci was −0.077). After the MLC corrections of AC, the estimates from all the MR methods were all positive and largely consistent (Fig. 4 and Supplementary Data 10). We also ran a reverse GSMR analysis to test the effect of BMI on AC without the MLC corrections and found a significant and negative effect of BMI on AC ($\hat b_{BMI \to {\mathrm{AC}}} = - 0.076,\;P = 1.11 \times 10^{ - 33}$) (Supplementary Data 11), consistent with the observation above that high BMI might be one of the reasons to reduce AC (Table 1).

In addition to the UKB data, we also analysed GWAS summary data for AC from Liu et al.^5^ with a sample size of ~1 million consisting of ~42.9% of the sample from 23andMe and ~33.0% from the UKB (Methods). The results were similar to those from the analyses above using the AC GWAS data from the UKB only (Supplementary Data 10 and Supplementary Figs. 13–14), which implies that MLC may not be UKB-specific but also exist in other data sets because otherwise the biases would be smaller in this analysis given only one-third of the AC data were from the UKB. We further confirmed the biases in MR analyses from MLC by simulation (Supplementary Figs. 6 and 8), and demonstrated that the estimates of causal effects from MR were nearly unbiased after the MLC corrections (Supplementary Fig. 9).

### The J-shaped relationship between AC and CVD

In epidemiological studies, there are debates about whether moderate drinking is protective against CVD because of an observed J-shaped relationship between AC and CVD^2,19,51^. We showed that the moderate drinking group (0 < AC ≤ 25 grams/week), often used as the reference group to compute the effect (odds ratio, OR) of AC on disease risk, was enriched with individuals from the LESS group which had a higher CVD incidence than the SAME and MORE groups (Supplementary Figure 15). This could result in a higher CVD incidence in the reference group than average, leading to a J-shaped relationship between AC and CVD (Supplementary Fig. 16a). Although the J-shaped relationship between AC and CVD did not change much after the MLC corrections (Supplementary Fig. 16b), it became monotonically increasing after excluding the LESS group from the reference (Supplementary Fig. 16c). Polygenic predictor of AC showed no evidence for any protective effect of moderate drinking against CVD (Supplementary Fig. 16d), consistent with the result from a previous study^51^. We ran a simulation to confirm that if the true relationship between two traits X and Y is a J-shape curve, then the relationship between the genetic predictor of X and Y is expected to be J-shaped (Supplementary Fig. 17). Our results indicated that the J-shaped relationship between AC and CVD observed in epidemiological studies might be driven by longitudinal changes due to disease ascertainment (Supplementary Note 3).

### Biases from MLC in other self-reported behavioural traits

Self-reported smoking data in the UKB is also likely to suffer from MLC. Similar to that for AC, all the current smokers were asked “Compared to 10 years ago do you smoke less/the same/more nowadays?”. We partitioned the current smokers (*n* = 32,801) into the LESS, SAME, and MORE groups (Supplementary Note 4). The LESS group had a higher disease count (1.69, s.e.m. = 0.01) than the SAME group (1.56, s.e.m. = 0.01) but a lower disease count than the MORE group (1.84, s.e.m. = 0.03) (Supplementary Data 12); these results were different from those observed in AC (see below for more discussion). In the LESS group, individuals who had reduced CPD because of illness or doctor’s advice had a much higher mean disease count (2.73, s.e.m. = 0.03) compared to the entire sample (1.45, s.e.m. = 0.002) or all current smokers (1.66, s.e.m. = 0.01), indicating that smoking intensity was also ascertained by disease burden. However, unlike AC, the $\hat r_g$ between CPD and common diseases were mostly consistent across the LESS, SAME and MORE groups (Supplementary Fig. 18), and there were negligible differences between the $\hat r_g$ estimated using the CPD GWAS data of the whole sample before and after correcting for MLC (Methods; Supplementary Data 13 and Supplementary Fig. 18).

Finally, we investigated physical activities (PA) in the UKB. The PA traits included self-reported METT (Metabolic Equivalent Task in Total) scores, IPAQ (International Physical Activity Questionnaires), and overall acceleration average (OAA, measured by wrist-worn accelerometers). We investigated these three measures in this study because they are the most commonly used PA indicators and available in the UKB. IPAQ is a derived categorical trait (low, moderate, and high) that utilises information from the METT and its three subsets: walking, moderate, and vigorous activities (Methods). We first estimated the $r_g$ between METT, IPAQ and OAA and between METT from the three IPAQ subgroups (Supplementary Fig. 19). We found a significant genetic heterogeneity between METT and IPAQ ($\hat r_g = 0.795$, s.e. = 0.016) and a small genetic overlap of either METT or IPAQ with OAA ($\hat r_g = 0.232$ with s.e. = 0.037 for METT and $\hat r_g = 0.390$ with s.e. = 0.034 for IPAQ). We then estimated the $r_g$ between PA and 18 common diseases. While the $\hat r_g$ of IPAQ and OAA with the diseases were mostly negative, METT showed positive $\hat r_g$ with most diseases (Supplementary Data 15 and Supplementary Fig. 20). It was also found that the $\hat r_g$ of METT from the low IPAQ subgroup with the diseases were highly consistent with those of IPAQ and OAA but mostly in the opposite direction to those of METT from the moderate and high IPAQ subgroups (Supplementary Fig. 20), indicating potential biases in METT from the moderate and high IPAQ subgroups, in line with the finding from a previous study^52^. In addition, the phenotypic correlation between the first and third assessment (*n* = 11,484) of METT was only 0.431, implying substantial longitudinal changes. Unfortunately, these changes were undocumented for the majority of UKB participants, so we were not able to perform correction as we did for smoking and drinking. It is known from prior work that device-measured PA show consistent inverse relationship with BMI, blood pressure, and adiposity^53^, while self-reported PA show inconsistent genetic correlation patterns at different intensity levels^54^, and that self-reported records in elder cohorts could suffer more from recall bias due to the high proportion of cognitive impairment^55^. Together with the evidence from the literature, our results suggest that IPAQ and OAA are better PA indicators than METT in the UKB, and that the $r_g$ estimates for METT are likely to be biased by disease ascertainment.

## Discussion

In this study, we raised concerns that genetic analyses of human behavioural traits could be biased by misreports and longitudinal changes. AC in the UKB was used as the main example to demonstrate the detrimental effects of MLC on several genetic analyses commonly used to identify variant-trait associations or estimate the genetic or causative relationship between traits. Our results showed that disease ascertainment was likely to be the main cause of the MLC biases, which can be largely corrected for using additional information (e.g., intake frequency and medical records) and coarse longitudinal data (e.g., self-reported longitudinal changes). Our results also showed that the MLC corrections proposed in this study added value to the routine quality controls (QC) in GWAS for behavioural traits. Additionally, biases due to longitudinal changes appeared to be larger than that due to misreports, because the longitudinal changes were observed in more than half of the participants, while misreports only accounted for 10~20% of the UKB sample (at least for those we have identified thus far), as verified in our simulations (Supplementary Note 2 and Supplementary Figs. 5–8).

Our findings provide a plausible explanation for the long-standing controversy about the effects of AC on health outcomes in genetic^4,5^ and epidemiological studies^2,3,19^. While it seems that most inconsistent estimates in previous studies were due to MLC, there are several reasons why some studies suffered from stronger biases than others. First, the average AC level varied across data sets (from 2.9 to 19.3 units/week across 24 studies)^5^, suggesting heterogeneity in drinking behaviours among different regions or populations. Second, since we have demonstrated that biases from MLC were mainly attributable to disease ascertainment, different disease prevalence between populations may lead to different patterns of MLC. Third, as MLC are associated with other factors, such as SES, studies with ascertainment of any of the MLC-associated factors would lead to a change of the MLC pattern. Last but not least, the pattern of MLC could vary in different age groups. For instance, disease ascertainment is expected to have a larger influence in middle-aged populations than in younger populations because younger populations are less likely to be affected by common diseases investigated in this study^56^. This is supported by the observation that the older UKB participants had a higher mean disease count with a higher proportion of them reducing AC due to illness or doctor’s advice (Supplementary Fig. 21). To account for a potentially non-linear relationship between AC and age, we fitted age squared as an additional covariate in the AC GWAS but observed little difference in the estimates of genetic or causal association between AC and BMI (Supplementary Fig. 22).

The MLC biases could differ for different behavioural traits such as AC and CPD. The main reason for the difference in the MLC bias pattern between AC and CPD is likely to be that the participants in the LESS group had a much higher mean disease count than those in both the SAME and MORE groups for AC (Table 1), indicating strong disease ascertainment, whereas such disease ascertainment was not apparent for CPD, e.g., the mean disease count in the LESS group is lower than that in the MORE group (Supplementary Data 12). More specifically, in the LESS group for CPD, the illness subgroup (i.e., participants reduced CPD because of illness) has a higher mean CPD level than the other subgroups (Supplementary Data 12), whereas in the LESS group for AC, the illness subgroup has a lower mean AC level (7.33 units/week) than the other subgroups (8.63 units/week). We hypothesise that these differences are because the likelihood of whether people choose to stop or reduce smoking due to reasons such as illness is different from that for drinking, e.g., when affected by illness, people tend to quit rather than reduce smoking but tend to reduce rather than stop drinking. This hypothesis is supported by the observations in the UKB that ~77% of the ever smokers are former smokers (Supplementary Data 14) while only ~3% of the ever drinkers are former drinkers (Supplementary Data 1).

Our study certainly has limitations as it is almost impossible to correct for all the biases with limited availability of relevant data. First, the 9,064 individuals who were suspected to underreport their AC are very likely to be only a subset of all the underreporting individuals. Thus, more effective methods are needed to identify the remaining underreporting individuals. Second, there are many reasons for MLC. These reasons include the self-reported reasons such as illness, doctor’s advice, health precaution and financial issues, and other reasons such as social desirability, major life changes (e.g., change of marital status and having a child), influences from family members or friends, religious experience, self‐evaluation and legal problem^57,58^. In the UKB survey, ~58% of the individuals with reduced alcohol intake reported that the reduction was due to “other reasons” or “do not know” in the survey (Table 2). Any of the reasons especially those related to disease and health precaution, if not accounted for, would lead to biases in GWAS and subsequent analyses. Also, since social acceptance is an important factor for the MLC reasons, the change of social acceptance over time might give rise to differences in MLC between real-time and retrospective reports. Third, some participants may have misreported their longitudinal changes, giving rise to an incorrect classification of longitudinal change groups. Fourth, the coarse longitudinal change information itself is cross-sectional (10 years before the time point of the first assessment), meaning that some of the changes that occurred beyond the time frame might not be accounted for in this study. Fourth, 15% of the current drinkers who did not report their AC level were removed from the analysis. One solution, as implemented in a previous study^59^, was to impute the missing values based on intake frequency and gender. However, 99.8% of the unresponsive individuals in the UKB were occasional drinkers while only 9.4% of the responsive individuals were occasional drinkers, which might lead to a systematic heterogeneity between the observed and imputed data sets. Thus, imputation just based on self-reported intake frequency and gender could be problematic. Finally, there were large differences in male/female ratio between the three longitudinal change groups (1.22, 0.95, and 0.59 in the LESS, SAME and MORE groups, respectively), giving rise to differences in MLC biases between males and females (Supplementary Fig. 23). In the MLC correction procedure, we removed mean and variance differences between the sex groups for AC by standardising AC in females and males separately, which substantially reduced the sex-differential MLC biases (Supplementary Fig. 23). However, if some of the trait-associated alleles are more or less frequent in one gender group^60^ and there are genotype-sex interaction effects, such locus-specific sex-differential biases are unlikely to be eliminated by our MLC corrections.

In conclusion, we advise awareness of the pitfalls when analysing data on behavioural traits in biobank data sets such as the UKB. Misreports and longitudinal changes of behavioural traits by disease ascertainment could create biases and thereby induce spurious signals and a loss of power in GWAS. Biases in GWAS summary statistics due to MLC could further lead to biased estimates in follow-up analyses such as genetic correlation and Mendelian randomisation. As more biobank data sets have become accessible, it is important to identify, investigate, and correct for these biases in all kinds of behavioural traits including smoking, drinking, diet, physical activity, sleep, and self-rated health status. A longitudinal study of 1 million individuals for several decades seems impractical at present, but we have shown that the biases in AC can be largely corrected for by phenotypic QC and longitudinal adjustment when additional phenotype information (intake frequency, medical records, longitudinal change, and reasons, etc.) are available. Questionnaires on lifetime use may provide more accurate estimates of the effects of behaviours on health outcomes at a much lower cost than a longitudinal follow-up study. Researchers should be more careful regarding these biases when conducting analyses for behavioural and other modifiable traits using biobank data sets with self-reported records.

## Methods

### Phenotypic data and quality controls

We obtained behavioural and disease traits from the UK Biobank (UKB) data^23^. The UK Biobank has approval from the North West Multicentre Research Ethics Committee (MREC), and informed consent has been obtained from all participants. There were 455,607 individuals of European ancestry with complete information on sex, age and principal components (PCs). The self-reported drinking statuses (data-field ID: 20117) were: never drinkers (*n* = 14,488), previous drinkers (*n* = 15,912), current drinkers (*n* = 424,507), and unknown (446 participants preferred not to answer and 254 provided no response). We removed “former drinkers” from all the analyses in this study, considering the occurrence of the “sick quitter phenomenon”^61^. Among the 424,507 current drinkers, 358,449 individuals reported their intake level. The AC level was summed up as a weekly total intake score (units/week) of all the alcoholic drink subtypes including beer plus cider, red wine, champagne plus white wine, spirits, and fortified wine. The mean of AC was 10.67 units per week (s.d. = 10.23). One unit was defined as one measure for spirits, one glass for red wine/white wine/champagne, or one pint of beer/cider. The raw AC units were transformed by log_2_ (raw AC units + 1) to avoid having a heavily skewed distribution. The smoking intensity was measured in cigarettes per day (CPD) in all current smokers (data-field ID: 3456; *n* = 32,801). Physical activity traits in the UKB were collected from both self-reported questionnaires and devices (wrist-worn accelerometers). METT is a total score of the Metabolic Equivalent Task (MET) minutes per week for walking, moderate activity, and vigorous activity (data-field IDs: 864, 874, 884, 894, 904, and 914; *n* = 417,938). IPAQ is a derived categorical trait that utilises the information from the METT and its three subsets mentioned above (see transformation criteria in https://biobank.ndph.ox.ac.uk/showcase/refer.cgi?id=540). The three IPAQ categories are denoted as low, moderate, and high ($n = 100,611$, 190,056, and 127,271, respectively). OAA (overall acceleration average) is an objective assessment of physical activity using a wrist-worn accelerometer (data-field ID: 90012; *n* = 97,006). The participants were voluntary, and the measurements were collected for seven consecutive days (see Doherty et al.^62^ for more details).

Following Zhu et al.^39^, we extracted phenotypic data of common diseases based on the primary (data-field: 41202) and secondary (data-field: 41204) ICD 10 codes and self-reported diseases (data-field: 20002) (Supplementary Data 4). There were 22 common diseases in total, and we further filtered out 4 diseases with a prevalence <2% in the UKB. The mean disease count was 1.45 (s.d. = 1.56) in the whole sample and 1.41 (s.d. = 1.53) in current drinkers. Body mass index (BMI) was obtained from the physical measurements (data-field: 21001). Educational attainment (EA) was indexed by years of school derived from qualification data (data-field ID: 6138). Household income (HI) was measured by the average total household income before tax (data-field ID: 738). For quantitative traits, extreme phenotypic values outside the mean ± 7 s.d. range in each sex group were excluded.

### Correcting for misreports and longitudinal changes

Our MLC corrections consist of two steps. The first step is a phenotypic quality control (QC) procedure used as an attempt to minimise the effects of misreports. We removed the individuals who self-reported as (1) never drinkers (*n* = 14,488), (2) current drinkers with reported weekly consumption of zero (*n* = 9064), and (3) current drinkers who provided no response to AC (*n* = 66,058), and retained a total of 349,385 individuals. The second step is to account for self-reported longitudinal changes compared to 10 years ago (data-field ID: 1628). We partitioned the individuals who passed the QC above into three groups based on the longitudinal change (i.e., LESS, SAME or MORE) and conducted GWAS within each group. In the LESS group, we further removed individuals who reduced their AC because of being ill or doctor’s advice, i.e., longitudinal change due to disease ascertainment (data-field ID: 2664; *n* = 11,886). We then performed an inverse-variance weighted meta-analysis of the GWAS results from the three groups (*n* = 336,469). This partitioning strategy efficiently removed any difference in mean or variance between the three groups. More details of the MLC corrections are shown in Supplementary Figs. 1 and 2.

### Genome-wide association analysis

The UKB genotype data were cleaned and imputed into the Haplotype Reference Consortium (HRC)^63^ panel by the UKB team^23^. We selected a subset of the sample of European ancestry (*n* = 456,426) from the whole UKB cohort by projecting the individuals onto the PCs from the 1000 Genome Project (1KGP). Genotype posterior probabilities were converted to hard-call genotypes using PLINK2 (–hard-call-thresh 0.1)^64^. We removed SNPs with a minor allele count <5, Hardy-Weinberg equilibrium test *P*-value < 1 × 10^−6^, missing genotype rate >5%, or imputation info score <0.3. For binary traits, we performed BOLT-LMM analysis^65^ with sex, age and the first 10 PCs fitted as covariates and then transformed the estimates of SNP effects on the observed 0-1 scale to odds ratios (OR) by LMOR^66^. For quantitative traits, we adjusted the phenotypic values for sex and age, standardised the adjusted phenotypes to z-scores, excluded individuals with $\left| z \right| \,> \,5$, and conducted the BOLT-LMM analysis^65^ with the first 10 PCs as fitted as covariates in the model.

Considering a loss of power due to decreased sample size by MLC corrections, we randomly down-sampled the GWAS data by 21,940 individuals and repeated this process 30 times. We used a z-statistic to test if the number of loci that became non-significant (or changed from non-significant to significant) after the MLC corrections is significantly different from that expected by random down-sampling. The average number of loci that became non-significant due to down-sampling was 10.03 (standard error of the mean s.e.m. = 0.85), significantly ($P = 2.08 \times 10^{ - 12}$) smaller than the decrease in the number of genome-wide significant loci due to the MLC corrections (i.e., 16). For the GWAS signals lost because of down-sampling, the average proportion of significant associations with the metabolic/cardiovascular traits in PheWAS v20190117 was 31.2% (s.e.m. = 3.8%), which was significantly lower than the observed 44.9% ($P = 3.61 \,\times 10^{ - 4}$), supporting the enrichment of the 16 loci in metabolic/cardiovascular traits (Supplementary Data 3). We also identified 10 loci that became genome-wide significant only after the MLC corrections (Supplementary Data 2). The down-sampling analysis showed that only 3.27 loci (s.e.m. = 0.52) would be expected by chance (Supplementary Data 3), indicating that most of the 10 loci were likely to be masked by MLC in the uncorrected GWAS.

### Estimating heritability and genetic correlation

We used the LD score regression^67^ (LDSC) to estimate SNP-based heritability for a trait and the bivariate-LDSC^35^ to estimate genetic correlation between traits using ~1.2 million SNPs in common with those in HapMap 3 (ref. ^68^). For the 234 traits for which we obtained GWAS summary data from LD Hub (http://ldsc.broadinstitute.org/ldhub/), the LDSC analyses were performed online in LD Hub^36^. Note that due to the restricted access to the full summary statistics of the 23andMe data sets, we did not perform the genetic correlation analysis for AC using the full GSCAN data^5^.

### Mendelian randomisation analysis

Mendelian randomisation (MR) is a method to estimate causal effect of an exposure on an outcome using instrumental variables (IVs) associated with the exposure^40,41^. MR assumes that the IVs are independent of possible confounders that may associate with both the exposure and outcome. Also, the IVs are assumed not to be associated with the outcome other than mediated through the exposure. However, in real data, these assumptions can be violated, leading to a biased estimate of the causal effect^69^. We performed MR analyses to test the causal effect of AC on BMI using IVW, Robust, MR-Egger, simple median, weighted median, mode, and Con-Mix implemented in the R package ‘MendelianRandomization’ v0.4.2, MR-PRESSO v1.0, MRMix v0.1.0 and RAPS v0.2 in R, and GSMR implemented in GCTA v1.91.8beta (http://cnsgenomics.com/software/gsmr/). The IVs were selected from a clumping analysis of the GWAS summary statistics in GCTA-GSMR (clumping criteria: window size = 1 Mb, $P = 5 \times 10^{ - 8}$ and LD $r^2 = 0.01$).

### Simulating data with disease ascertainment

We carried out simulations to mimic the bias due to disease ascertainment in GWAS and its follow-up analyses. If individuals who are affected by a disease tend to change a behaviour, such a change would lead to a spurious correlation between the disease and behaviour. We considered four scenarios in the simulation: (I) the disease liability (D) is independent of the behavioural trait (Y), and 100 SNPs are associated with Y only; (II) Y had a causal effect on D, and 100 SNPs are associated with Y (and D mediated through Y); (III) Y and D are independent, and 100 SNPs are associated with D only; (IV) Y had a causal effect on D, 100 SNPs affected Y (and D mediated through Y), and another set of 100 SNPs affected D directly. In each scenario, to mimic the disease ascertainment, we reduced the values of Y for the individuals who had high values of D. More specifically, if the D value of an individual passed a threshold (e.g., top 10%), the corresponding Y value would be subtracted by a constant. We set the disease ascertainment threshold to be 10%, 20%, 30%, or 40% and considered a subtraction from Y value by 1, 2, 3, 4 or 5 standard deviations. We then conducted GWAS and estimated SNP effect correlation between Y and D using the *r*_b_ approach developed in a previous study^70^ as well as the causal effect of Y on D using GSMR. To demonstrate the effectiveness of our MLC correction procedure in correcting for disease ascertainment bias, we divided the individuals into two groups (LESS and SAME) and then conducted the GWAS separately, followed by a meta-analysis. Details of the simulation process and parameter specifications can be found in Supplementary Note 2.

### Reporting summary

Further information on research design is available in the Nature Research Reporting Summary linked to this article.

## Supplementary information

Supplementary Information

Peer Review

Reporting Summary

Description of Additional Supplementary Files

Supplementary Data 1

Supplementary Data 2

Supplementary Data 3

Supplementary Data 4

Supplementary Data 5

Supplementary Data 6

Supplementary Data 7

Supplementary Data 8

Supplementary Data 9

Supplementary Data 10

Supplementary Data 11

Supplementary Data 12

Supplementary Data 13

Supplementary Data 14

Supplementary Data 15

## Data Availability

The GWAS summary data generated in this study are available at http://fastgwa.info/share/mlc-paper/. The individual-level genotype and phenotype data from the UKB are open to all bona fide researchers upon application (https://www.ukbiobank.ac.uk/principles-of-access/). The GWAS summary statistics in the PheWAS database can be downloaded at https://atlas.ctglab.nl/. The full download links of GWAS summary statistics in the LD Hub can be found in the Lookup Center after login in at http://ldsc.broadinstitute.org/. The 1000 Genome Project data can be downloaded at https://www.internationalgenome.org/data/.
