# Publisher Correction: Genome-wide analyses of behavioural traits are subject to bias by misreports and longitudinal changes

**DOI:** 10.1038/s41467-021-21294-1

**Published:** 2021-02-08

**Authors:** Angli Xue, Longda Jiang, Zhihong Zhu, Naomi R. Wray, Peter M. Visscher, Jian Zeng, Jian Yang

**Affiliations:** 1 Institute for Molecular Bioscience, The University of Queensland, Brisbane, QLD 4072 Australia; 2 Queensland Brain Institute, The University of Queensland, Brisbane, QLD 4072 Australia; 3 School of Life Sciences, Westlake University, Hangzhou, Zhejiang 310024 China; 4 Westlake Laboratory of Life Sciences and Biomedicine, Hangzhou, Zhejiang 310024 China

**Keywords:** Genetic association study, Population genetics, Quantitative trait, Risk factors

Correction to: *Nature Communications* 10.1038/s41467-020-20237-6, published online 7 January 2021.

The original version of this Article was updated shortly after publication to update the volume number. During the update to the volume, the Article incorrectly gave a publication date of 7 January 2021 and Article number of 6450 in the PDF and HTML version; the correct publication date should have been 12 January 2021 and the correct Article number should have been 20211.

This has now been corrected in both the PDF and HTML versions of the Article.

