# Supplementary material for: Genome-wide analyses of behavioural traits are subject to bias by misreports and longitudinal changes
Source: Nat Commun. 2021 Jan 7;12:6450. doi: 10.1038/s41467-020-20237-6 (PMC7804181; doi:10.1038/s41467-020-20237-6)
Supplement: Supplementary file 1 — Supplementary Information [file 41467_2020_20237_MOESM1_ESM.pdf]

Supplementary Information

Genome-wide analyses of behavioural traits are subject to bias by misreports and longitudinal changes

Xue et al.

Supplementary Notes

Supplementary Note 1: Identifying misreporting "never drinkers" in the UKB

Following Klatksy et al.[1], we attempted to identify "unreliable" self-reported never drinkers using follow-up questionnaires and medical records. The UKB had online follow-up questionnaires in 2017. There were 11 questions related to "alcohol use" in the "mental health" category (n = 157,365). We extracted the "frequency of drinking alcohol" (data-field ID: 20414) of 3,627 self-reported never drinkers in the first assessment (2006-2010), but 335 of them (~9.2%) reported that they were not never drinkers in this follow-up assessment (2017). Although these individuals could change drinking status after a few years, it is reasonable to question the reliability of their reported drinking status in the initial assessment. We also extracted the ICD 10 codes (data-field ID: 41202) of 14,488 self-reported never drinkers. People with diagnosed alcohol-related diseases were very likely to have misreported their drinking status. The diseases include E24.4: alcohol-induced pseudo-Cushing's syndrome, F10: mental and behavioural disorders due to use of alcohol, G31.2: degeneration of nervous system due to alcohol, G62.1: alcoholic polyneuropathy, G72.1: alcoholic myopathy, I42.6: alcoholic cardiomyopathy, K29.2: alcoholic gastritis, K70: alcoholic liver disease, K85.2: alcohol-induced acute pancreatitis, K86.0: alcohol-induced chronic pancreatitis, R78.0: finding of alcohol in blood, T51: toxic effect of alcohol, Z50.2: alcohol rehabilitation, and Z72.1: alcohol use. There were 77 individuals diagnosed with these diseases; thus, their self-reported drinking status was also likely to be unreliable.

Supplementary Note 2: Simulation

To validate our findings, we performed a series of simulations to mimic MLC due to disease ascertainment. There were four simulation scenarios, as shown in **Supplementary Figure 4**. We simulated 20,000 individuals and 100 causal variants affecting a behavioural phenotype (Y) and another set of independent 100 causal variants affecting the liability of a disease (D). Both Y and D were quantitative. The variance explained by the causal variants was 0.6 for both Y and D, *i.e.*, $h_Y^2 = h_D^2 = 0.6$. The SNP effects were randomly drawn from $\mathcal{N}(0,1)$. The causal effect (b_{xy}) of Y on D was set to 0.2.

We mimicked the disease ascertainment by reducing Y to a lower level if the corresponding D value was high. In other words, those individuals with high D values (located in the 10, 20, 30 or 40% upper tail of the distribution) were regarded as disease carriers, and their Y values were deducted by a constant (1-5 standard deviations, *s.d.*). After the ascertainment, we rescaled Y and conducted GWAS for Y and D, and then estimated the correlation of true SNP effects (r_b) between Y and D accounting

for errors in estimated SNP effects using a recently developed approach[2], and the causal effect (b_{xy}) of Y on D using Mendelian Randomization (MR).

In model I, where Y and D were independent, and the SNPs were associated with Y only, the r_b and b_{xy} estimates were expected to be 0 in the absence of ascertainment, consistent with our simulation results (**Supplementary Figure 5a**). However, the ascertainment generated a negative correlation between Y and D, leading to negative estimates of both r_b and b_{xy} (**Supplementary Figures 5-6**).

In model II, where Y had a causal effect on D, and the SNPs only had direct effects on Y, the $\hat{r}_b$ only slightly decreased with the increased strength of ascertainment, suggesting that the SNP effect correlation estimate under a causal model was not heavily biased by the ascertainment (**Supplementary Figure 5b**). Even when 10% of the individuals in the upper tail of the distribution of D were reduced by 5 *s.d.* units in Y, the $\hat{r}_b$ only decreased from 1.000 (*s.e.* = 0.003) to 0.929 (*s.e.* = 0.003). In the meanwhile, the causal effect estimated from MR analysis increased from 0.200 (*s.e.* = 0.002) to 0.390 (*s.e.* = 0.004). Notably, the number of index SNPs decreased as the ascertainment became stronger (**Supplementary Figure 6b**), indicating that the ascertainment could reduce the power to detect causal variants in GWAS.

In model III, where Y and D were independent, and the SNPs were associated with D only, the ascertainment induced a negative correlation between Y and D (**Supplementary Figure 5c**), and more genome-wide significant SNPs were detected to be associated with Y as the ascertainment strength became larger (**Supplementary Figure 6c**).

In model IV, where Y had a causal effect on D with 100 SNPs affecting Y and another set of 100 SNPs affecting D, the $\hat{r}_b$ gradually changed from positive to negative as the ascertainment became stronger (**Supplementary Figure 5d**). In the MR analysis, when the ascertainment strength was modest, the $\hat{b}_{xy}$ was more robust than the $\hat{r}_b$ (**Supplementary Figure 6d**).

The simulations above are all for longitudinal change; however, we can also simulate underreporting using a similar procedure, *i.e.*, assigning a lower value to Y for individuals with large D. The only difference between underreporting and longitudinal change in the simulation was the proportion of individuals affected. We set the proportion of underreporting individuals from 2% to 8% of the upper tail of the distribution of D based on that observed in the UKB. Our simulation results showed that the effects of ascertainment bias from underreporting were smaller than those from longitudinal change (**Supplementary Figures 7-8**).

Supplementary Note 3: The relationship between AC and cardiovascular disease (CVD)

To investigate the observed relationship between AC and CVD, we first performed logistic regression analyses of cardiovascular disease on different AC intake levels as suggested in Wood et al.[3]. The relationship was J-shaped where moderate drinking showed a lower disease risk and heavy drinking showed a higher disease risk than that in the reference group ($0 < AC \leq 25$ grams/week) (**Supplementary Figure 15a**). We performed the MLC corrections by excluding underreporting individuals and individuals who reduced drinking because of illness or doctor's advice, and fitted longitudinal change as the covariate in the logistic model. The J-shaped relationship remained but the risk threshold (the point at which odds ratio, i.e. OR, of CVD becomes larger than 1 as AC increases) shrank towards the left (**Supplementary Figure 15b**). However, when we removed only the individuals who had reduced their drinking amount in the reference group, the relationship between AC and CVD became monotonically increasing (**Supplementary Figure 15c**), suggesting an enrichment of disease ascertained individuals in the reference group as demonstrated in **Supplementary Figure 14**.

We performed a simulation to verify whether we would expect a J curve between the genetic predicted of X and the raw phenotype of Y if the true relationship is a J curve. We first simulated X and Y in 50,000 unrelated individuals. There were 160 causal variants for X (total $h^2 = 0.3$), 100 causal variants for Y (total $h^2 = 0.3$), and 40 pleiotropic variants for X and Y (total $h^2 = 0.1$ for both X and Y). The SNP effects were randomly drawn from $\mathcal{N}(0,1)$. We simulated a J-shaped causal effect of X on Y (formula: $Y \sim X^2 + X$). Individuals with the top 20% Y values were regarded as disease carriers. We divided X into ten deciles and labelled the lowest 10% as the reference group and estimated the effect of X and Y using the rest nine deciles against the reference group in a logistic regression. We plotted the mean of X in each decile against the OR in each comparison as we did in the real data and observed a J-shaped curve (**Supplementary Figure 16a**). Then we used genome-wide significant variants ($P < 5 \times 10^{-8}$) for X to generate a genetic predictor of X, and then estimated the effect of the genetic predictor of X on the Y in different quantiles of X. The simulation was replicated 50 times and the relationship was still a J curve (**Supplementary Figure 16b**), which suggests we would expect a J curve between a genetic predictor of X and Y if the true relationship is a J curve.

Supplementary Note 4: MLC corrections for smoking intensity

According to the self-reported records in the UKB (data-field ID: 20116), there were ~245,000 never smokers, ~162,000 previous smokers and ~47,000 current smokers. The cigarettes per day (CPD) data were collected among the current smokers who used manufactured cigarettes or hand-rolled cigarettes (data-field ID: 3456). According to the self-reported longitudinal change information from 32,801 current cigarette smokers (data-field ID: 3506), 5,559 individuals increased their smoking intensity,

13,235 maintained the same intensity and 13,941 reduced their smoking intensity compared to 10 years ago (**Supplementary Data 12a**). We performed the MLC corrections for CPD by 1) partitioned the current smokers into three longitudinal change groups, 2) excluded 3,061 individuals who chose illness or doctor's advice as the reason for reducing smoking (data-field ID: 6158), 3) performed GWAS in each group with standardised CPD and meta-analysed GWAS summary statistics from the three groups. We compared the GWAS results for CPD with or without the MLC corrections (**Methods**) and found that the estimate of genetic correlation between CPD before and after the MLC corrections was not significantly different from 1 ($\hat{r}_g = 0.985, s.e. = 0.015$). Additionally, we did not observe any large differences in the $\hat{r}_g$ of CPD with diseases before and after the MLC corrections (**Supplementary Data 13** and **Supplementary Figure 17**).

Supplementary Figures

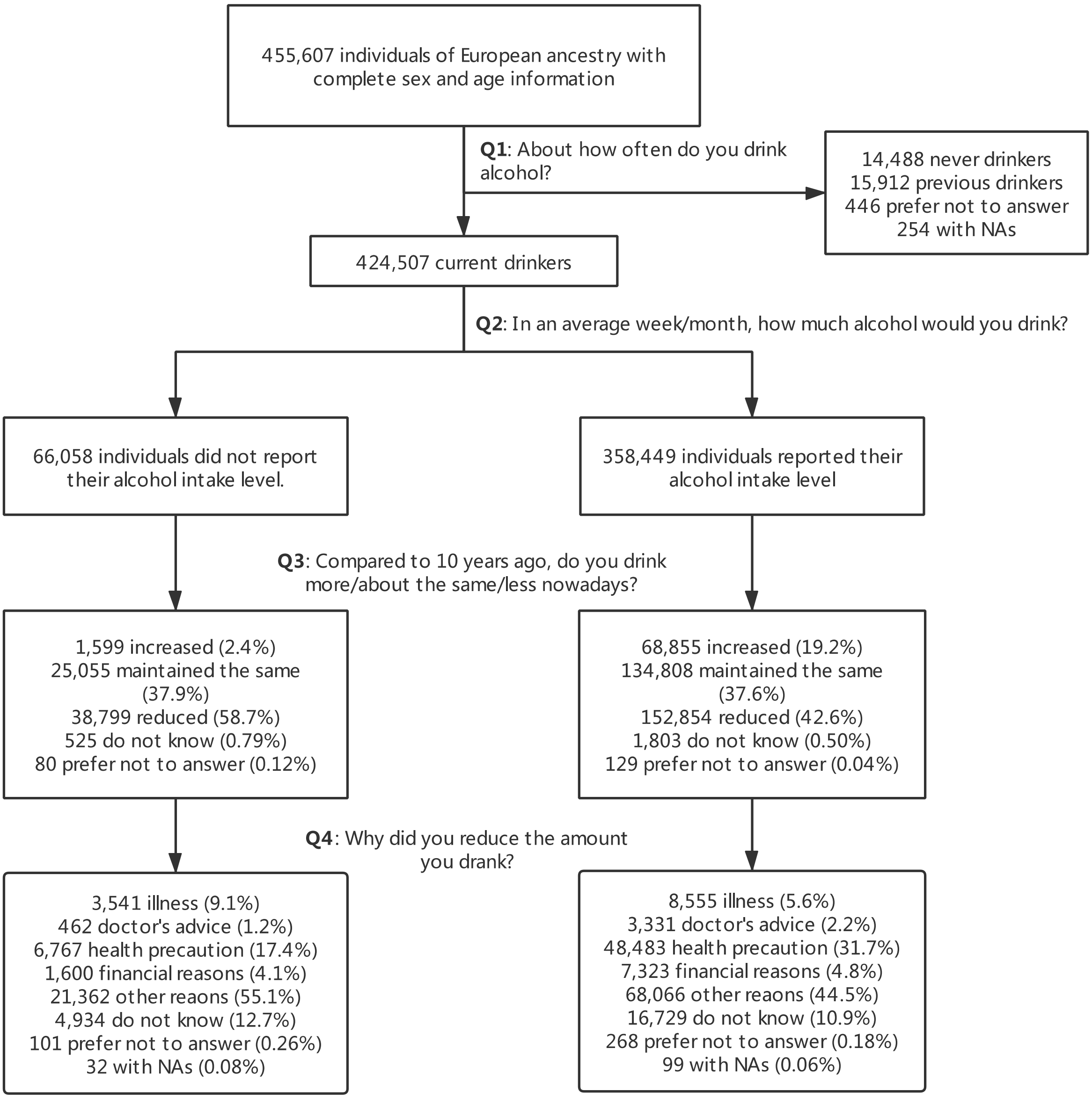

Supplementary Figure 1. Flow chart of the alcohol-related questionnaire in the UK Biobank. The full questionnaire can be found in pages 35-38 at http://biobank.ndph.ox.ac.uk/showcase/showcase/docs/TouchscreenQuestionsMainFinal.pdf.

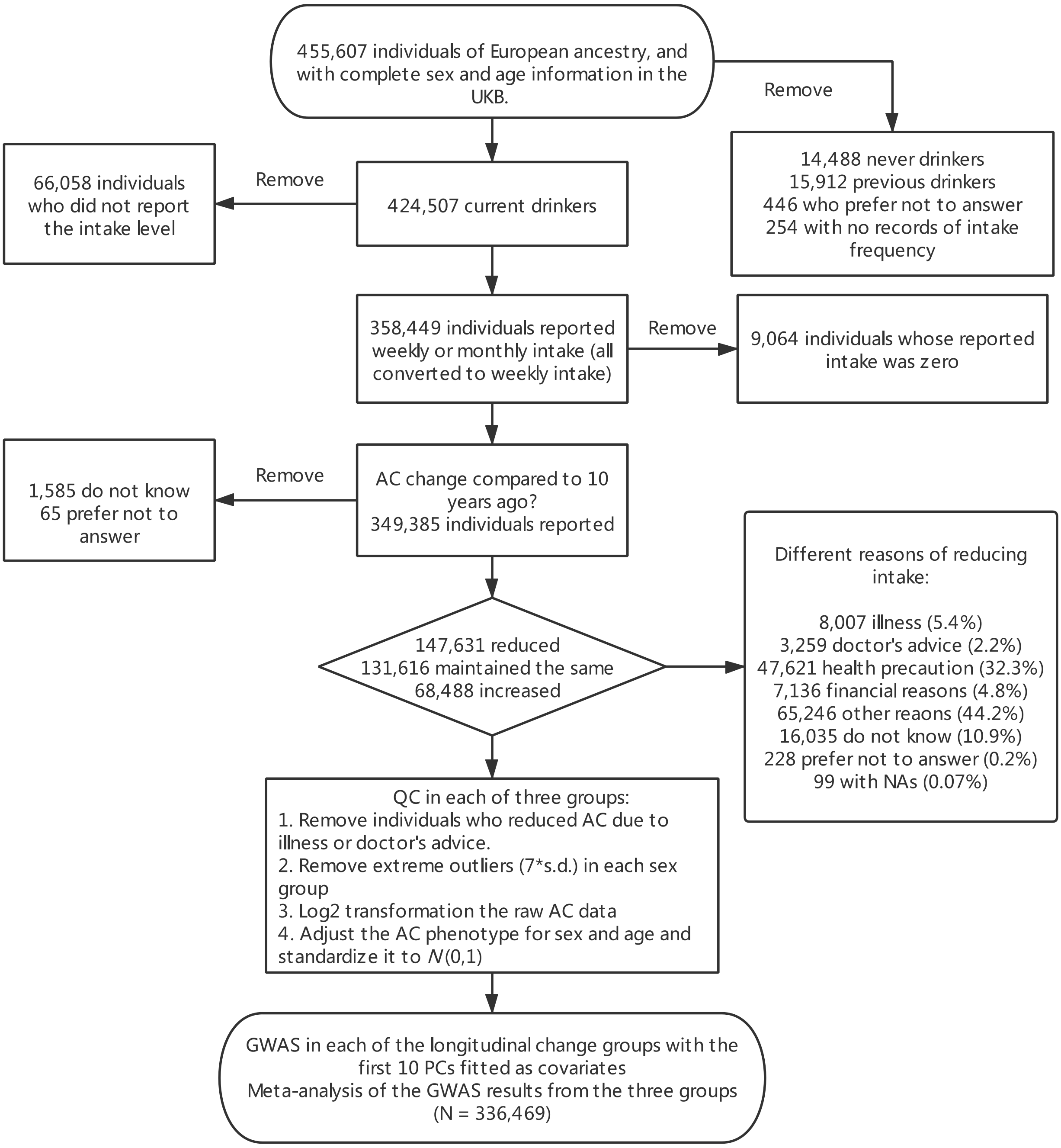

Supplementary Figure 2. Flow chart of the MLC corrections for alcohol consumption. UKB: UK Biobank. AC: alcohol consumption. QC: quality control. PC: principal component.

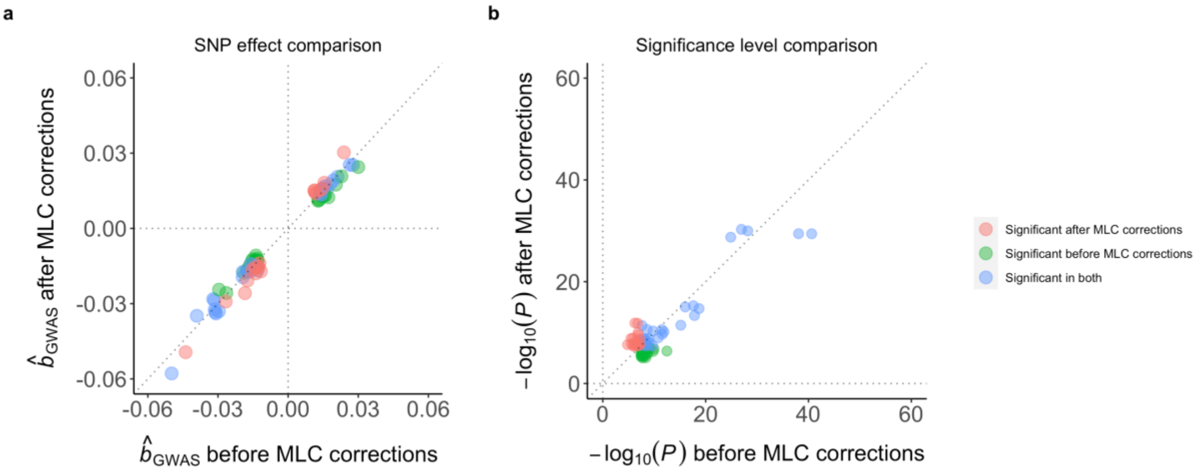

Supplementary Figure 3. Comparison between alcohol consumption GWAS results before and after the MLC corrections. (a): Effects of the AC-associated SNPs before and after the MLC corrections. The red dots denote the SNPs that were not significantly associated with AC but became significant ($P < 5 \times 10^{-8}$) after the MLC corrections. The green dots denote the SNPs that were significant but became non-significant the MLC corrections. The blue dots indicate the SNPs that were significant in both. (b): The -log10 *P*-values of the AC-associated SNPs before and after the MLC corrections. The top SNP rs1229984 at the *ADH1B* locus is omitted due to its large effect size; the effect of the T allele was -0.24 ($P = 4.10 \times 10^{-214}$) and -0.23 ($P = 1.04 \times 10^{-167}$), respectively, before and after the MLC corrections. The *P*-value indicates the GWAS significant level of each SNP with AC from BOLT-LMM analysis (two-sided χ^2test).

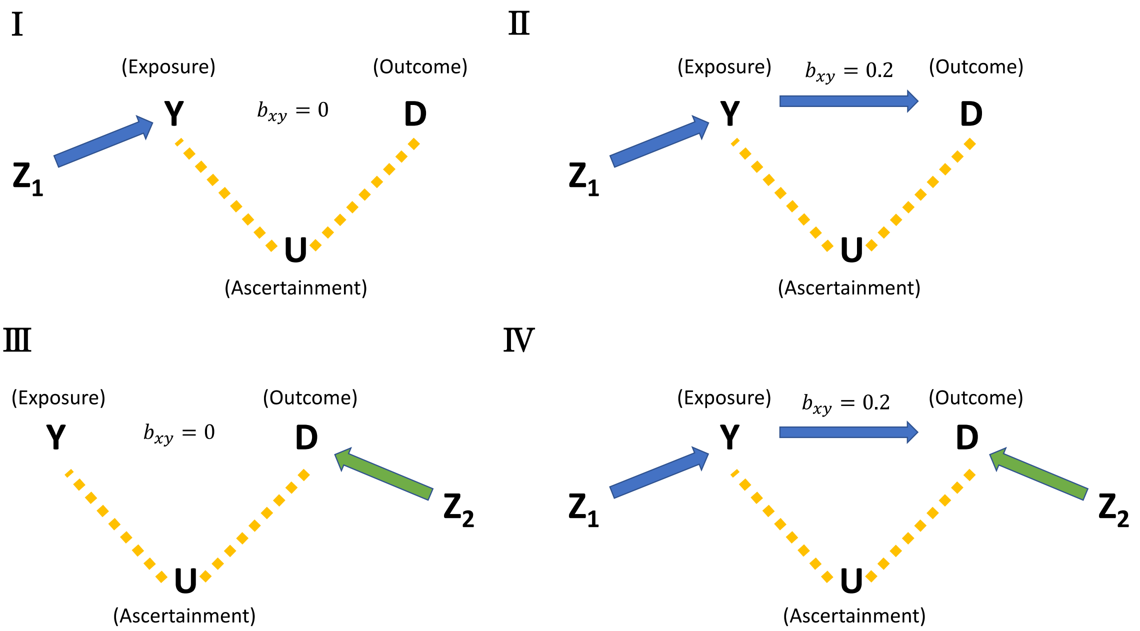

Supplementary Figure 4. Four models used in the simulations to mimic disease ascertainment. Y is a behavioural phenotype, D is the liability of a disease, Z_1 is a set of causal variants only for Y, and Z_2 is a set of causal variants only for D. The yellow dashed line indicates the association between Y and D induced by the change of Y conditioning on D via ascertainment (U). Model I: Y and D are independent, and 100 SNPs are associated Y. Model **II**: Y had a causal effect on D, and 100 SNPs are associated with Y (and D mediated through Y). Model **III**: Y and D are independent, and 100 SNPs are associated with D. Model **IV**: Y had a causal effect on D, 100 SNPs are associated with Y (and D mediated through Y), and another set of 100 SNPs are associated with D directly.

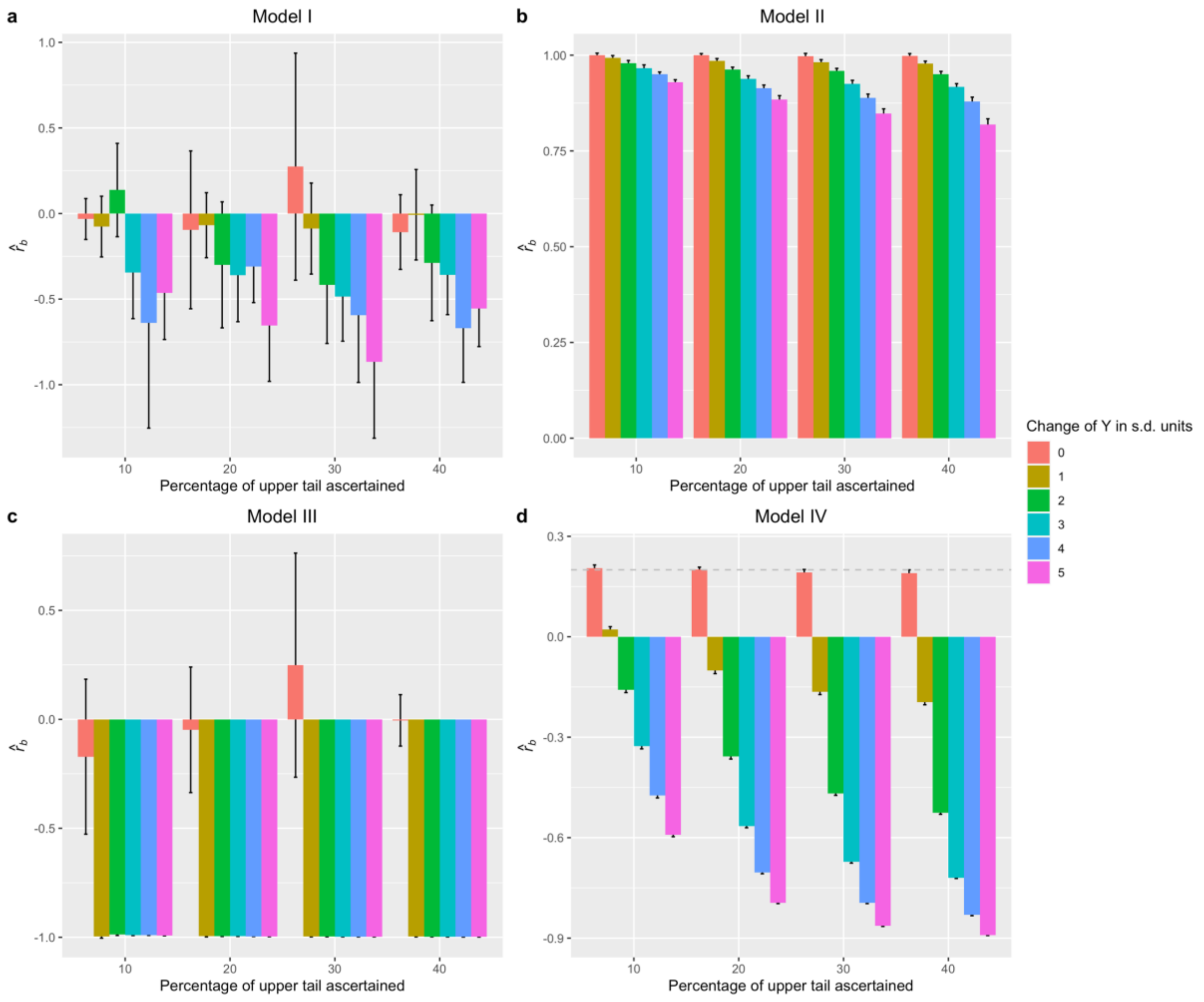

Supplementary Figure 5. Quantifying bias in the estimated SNP effect correlation due to longitudinal change by simulation. The four models are defined in **Supplementary Figure 4**. The x-axis indicates the percentage of ascertained individuals. The total sample size used in the simulation $n = 20{,}000$. The y-axis indicates the r_b estimates. The r_b is defined as Pearson's correlation between the effects of the genetic variants on Y and those on D accounting for errors in the estimated variant effects. The error bars indicate the 95% confidence interval of the r_b estimate. The colour of the bar indicates the strength of ascertainment (*i.e.*, the change of the phenotype Y in *s.d.* units). Change in *s.d.* = 0 means no ascertainment. The grey dashed line indicated $r_b = 0.2$.

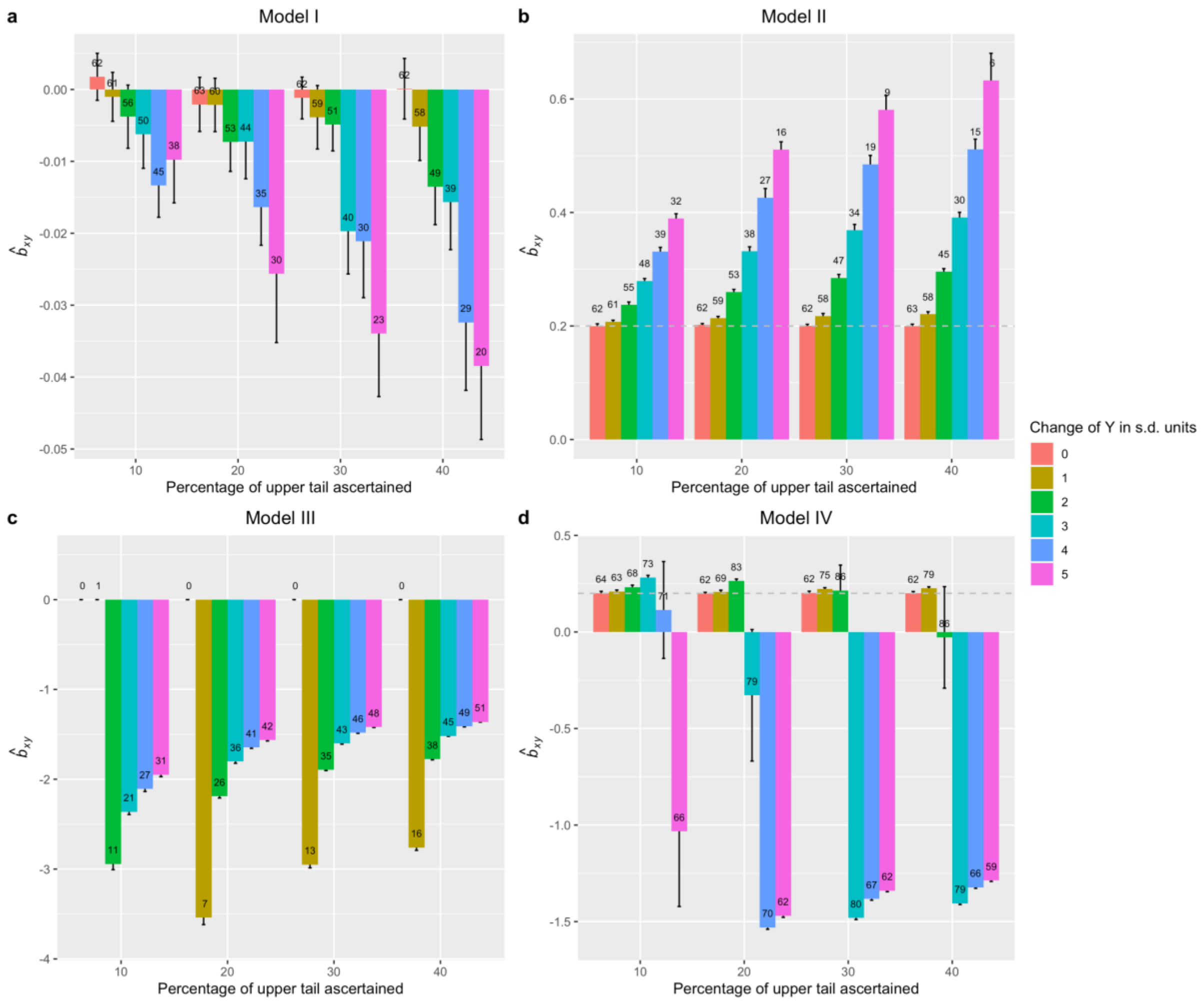

Supplementary Figure 6. Quantifying bias in the estimated causal effect due to longitudinal change by simulation. The four models are defined in **Supplementary Figure 4**. The x-axis indicates the percentage of ascertained individuals. The total sample size used in the simulation n = 20,000. The y-axis indicates the causal effect estimates, $\hat{b}_{xy}$. The error bars indicate the 95% confidence interval of the $\hat{b}_{xy}$. The colour of the bar indicates the strength of ascertainment (*i.e.*, the change of the phenotype Y in *s.d.* units). Change in *s.d.* = 0 means no ascertainment. The number labelled on the bar indicates the number of genome-wide significant SNPs of Y. Some of the bars are missing in panel C because there were not enough instrumental SNPs to perform the GSMR analysis. The grey dashed line indicated $b_{xy} = 0.2$.

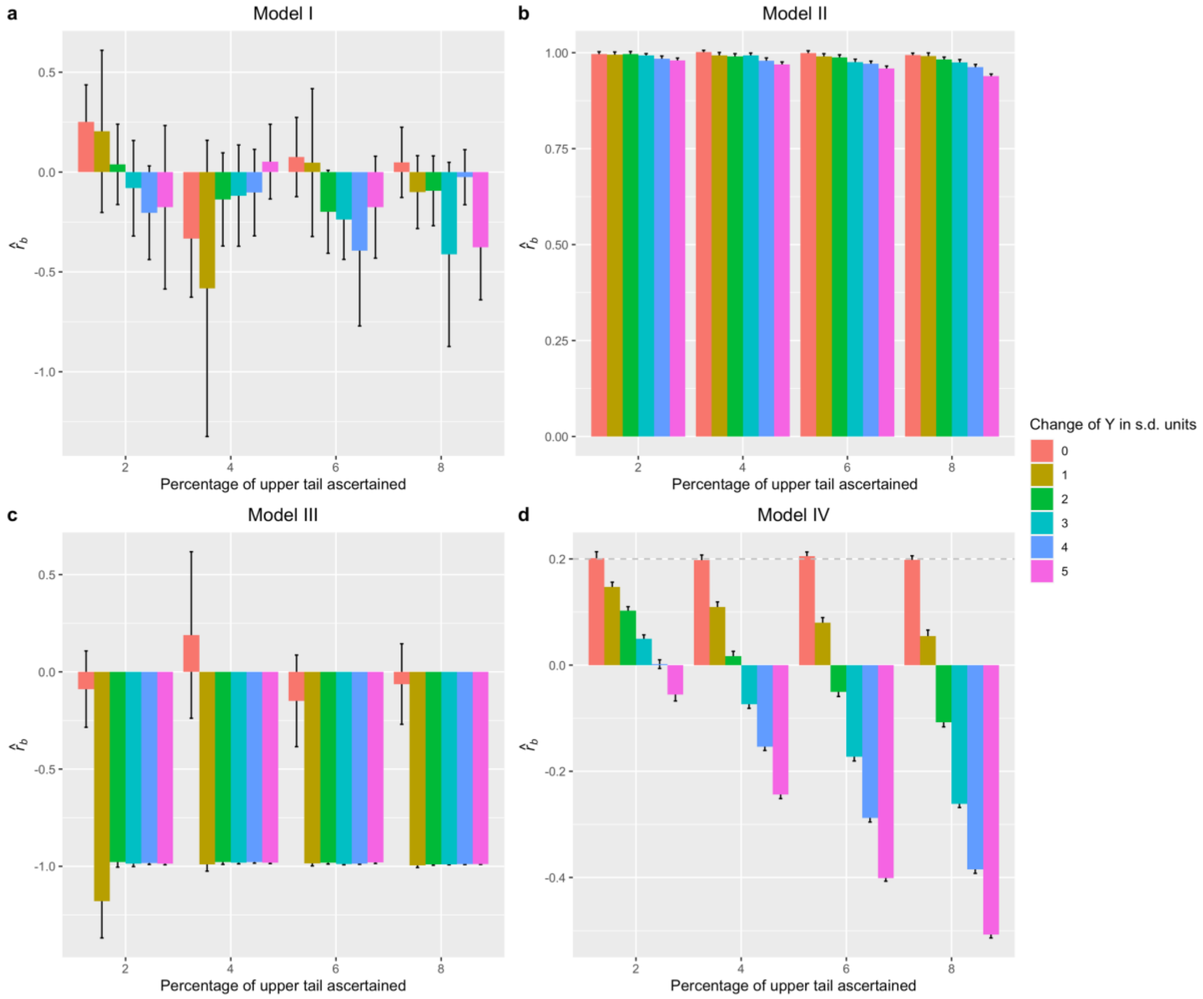

Supplementary Figure 7. Quantifying bias in the estimated SNP effect correlation due to misreporting by simulation. The total sample size used in the simulation $n = 20{,}000$. The error bars indicate the 95% confidence interval of the r_b estimates. All the labels and colour code are the same as those in **Supplementary Figure 5**.

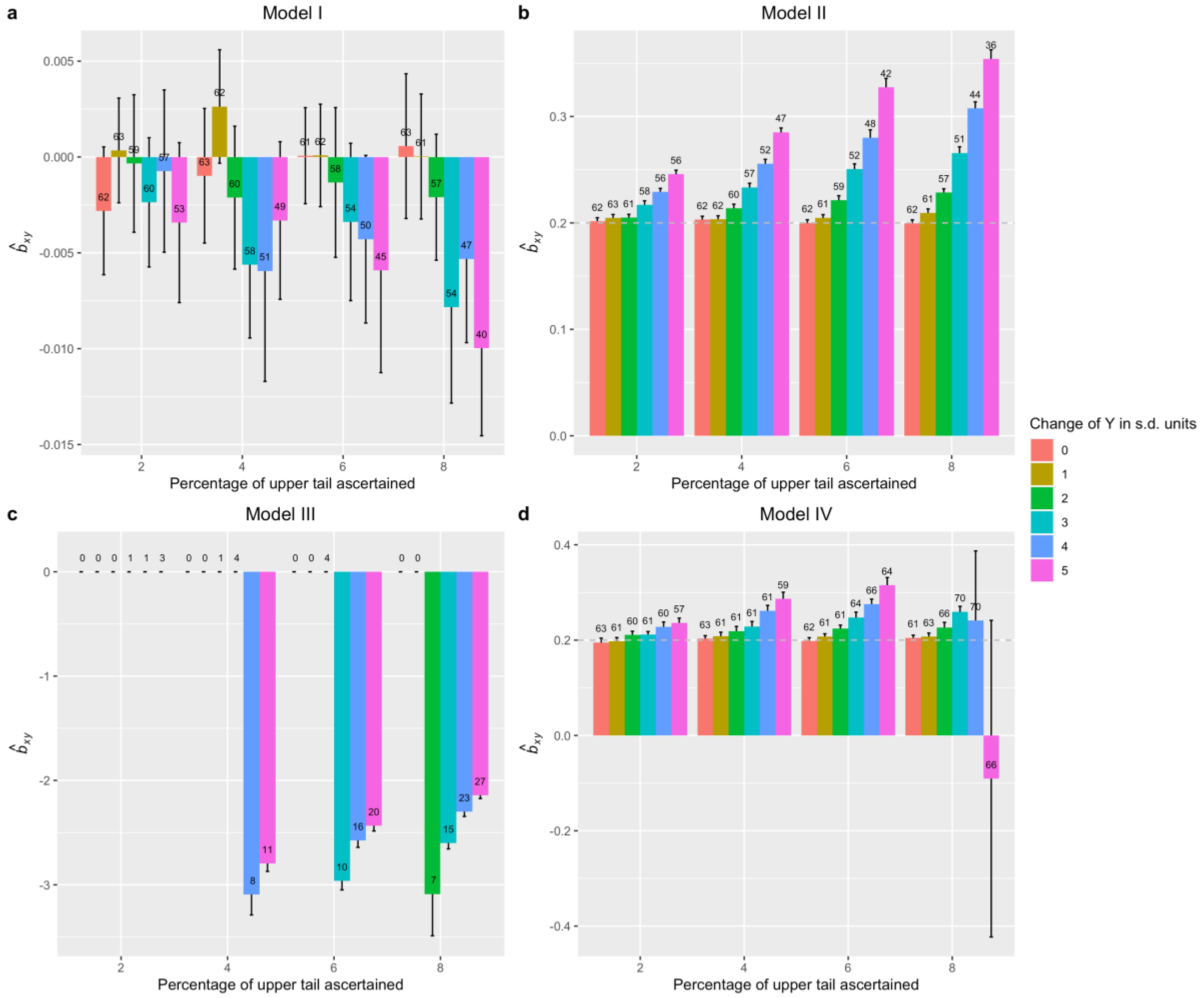

Supplementary Figure 8. Quantifying bias in the estimated causal effect due to misreporting by simulation. The total sample size used in the simulation n = 20,000. All the labels and colour code are the same as those in **Supplementary Figure 6**. The error bars indicate the 95% confidence interval of the $\hat{b}_{xy}$. Change in *s.d.* = 0 means no ascertainment. The number labelled on the bar indicates the number of genome-wide significant SNPs of Y. Some of the bars are missing in panel C because there were not enough instrumental SNPs to perform the GSMR analysis.

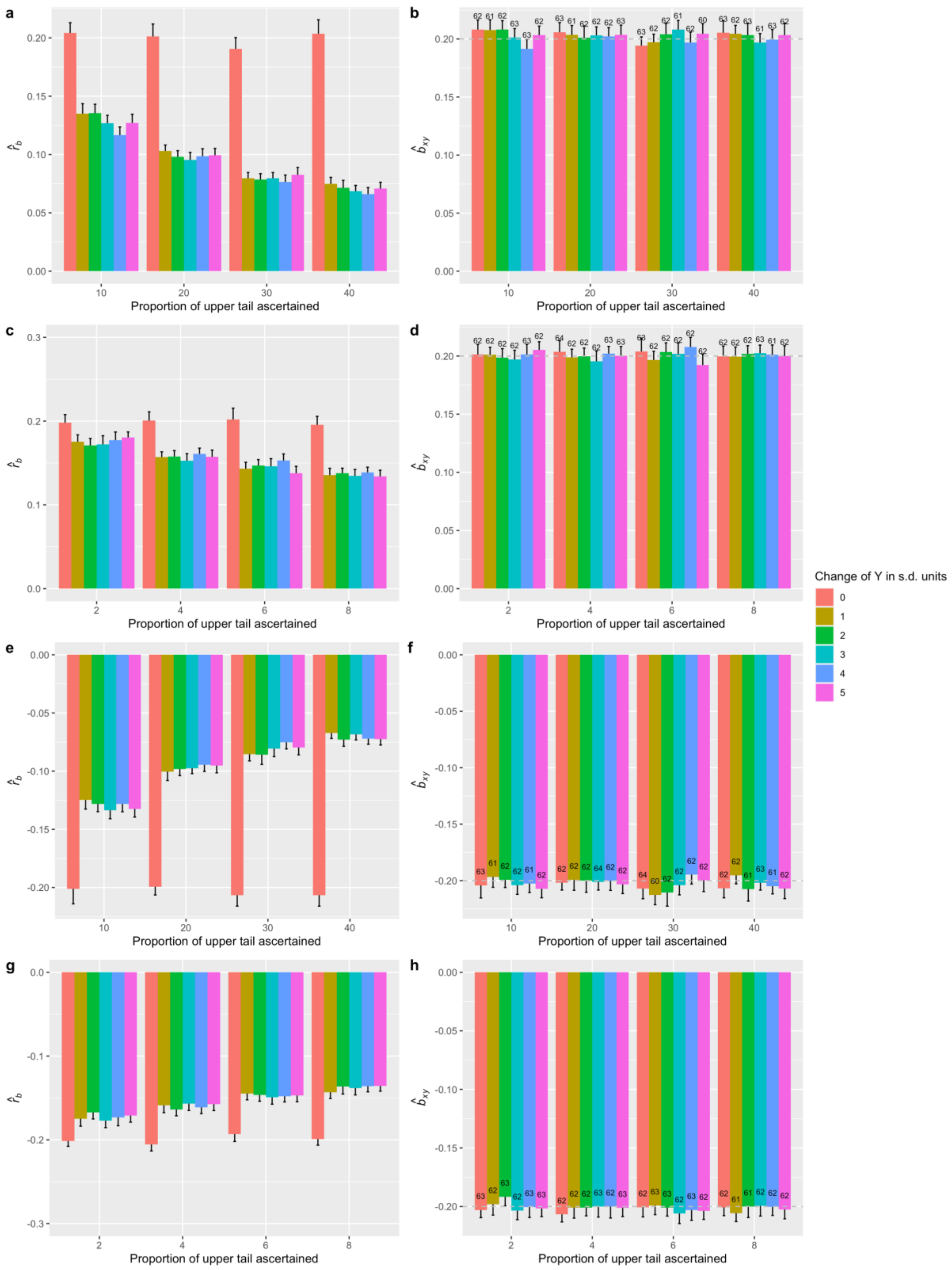
a
b
c
d
e
f
g
h
$\hat{r}_b$
$\hat{b}_{xy}$
Proportion of upper tail ascertained
Change of Y in s.d. units
0
1
2
3
4
5

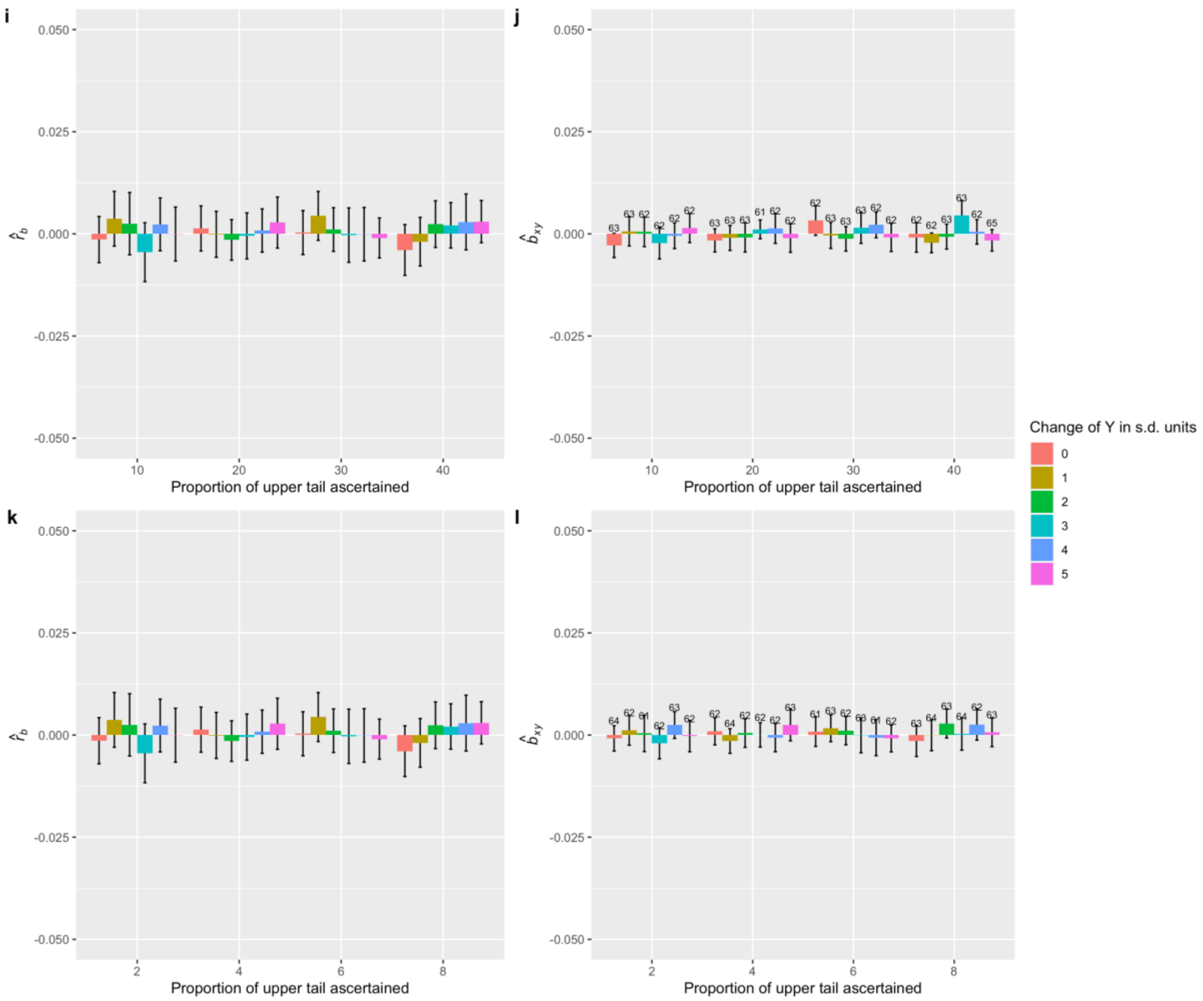

Supplementary Figure 9. Estimates of SNP effect correlation and causal effects in simulations after the MLC corrections. The total sample size used in the simulation n = 20,000. All the labels and colour code are the same as those in **Supplementary Figures 5** and **6**. Only the data simulated based on Model IV were analysed here. Panels (a) and (b) show the r_b and b_{xy} estimates after the MLC corrections in the presence of longitudinal change, respectively. Panels (c) and (d) show the r_b and b_{xy} estimates after the MLC corrections in the presence of underreporting, respectively. The error bars indicate the 95% confidence interval of the r_b or b_{xy} estimates. Panels (e) to (h) are based on the same simulation setting as those for panels A to D except for that b_{xy} is set to -0.2. Panels (i) to (l) are based on the same simulation settings as those for panels (a) to (d) except for that b_{xy} is set to 0.

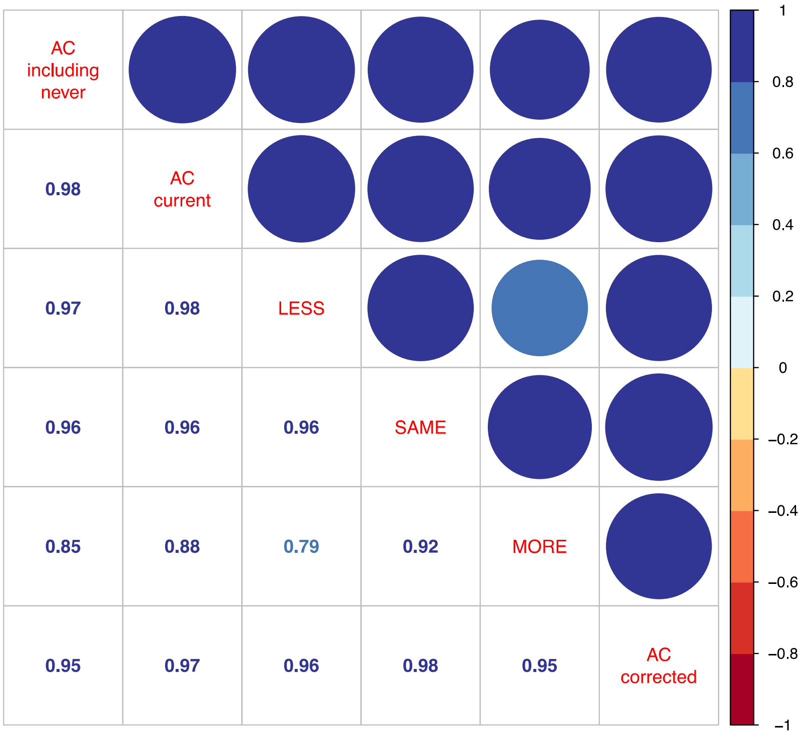

Supplementary Figure 10. Estimates of genetic correlation between different AC groups. The value in each cell below the diagonal denotes the r_g estimate from a bivariate LDSC analysis. The circle in each cell above the diagonal shows the r_g estimate visually: larger circle size and darker color indicate higher r_g estimate. "AC including never" represents alcohol consumption in current and never drinkers. "AC current" represents alcohol consumption in current drinkers. LESS, SAME, and MORE represent current drinkers whose AC levels were reduced, maintained the same, and increased, respectively, compared to 10 years ago. "AC corrected" represents alcohol consumption in current drinkers after the MLC corrections.

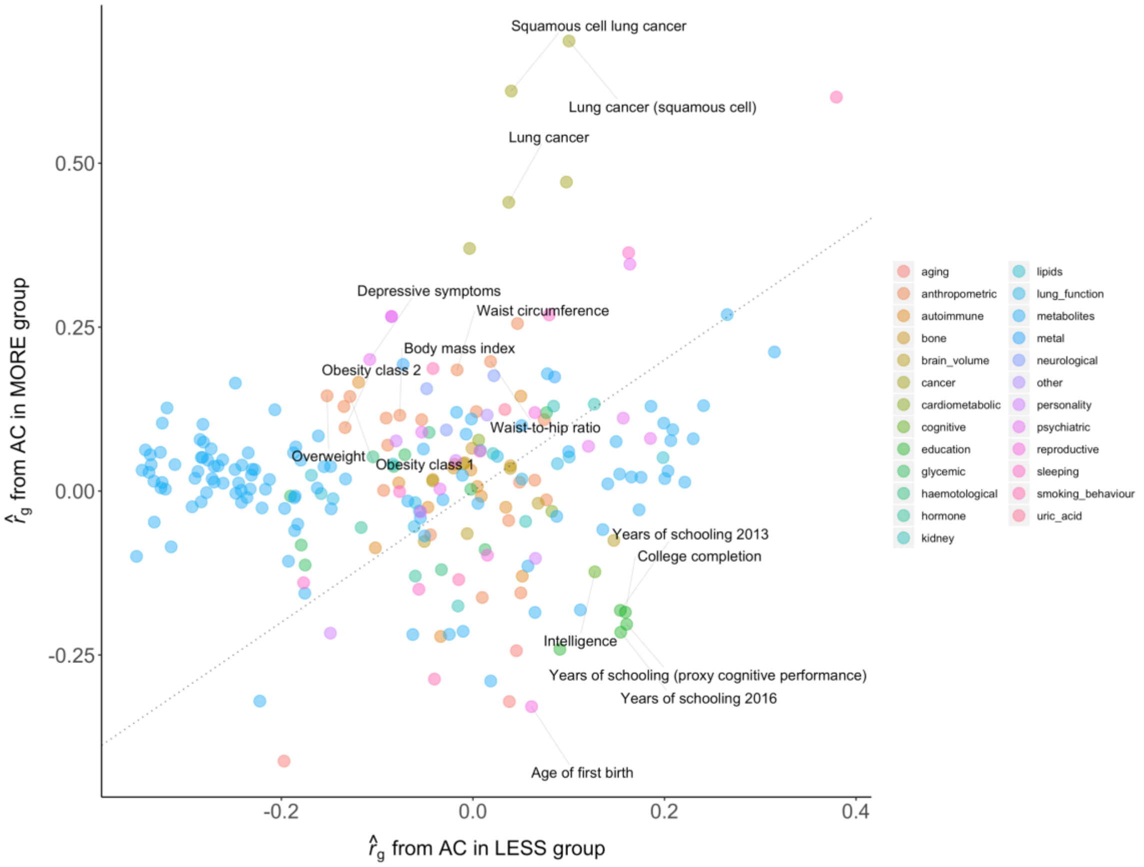

Supplementary Figure 11. Estimates of genetic correlation between AC and 234 traits in LD Hub. The x-axis indicates the r_g estimates using AC from the LESS group, and the y-axis indicates the r_g estimates using AC from the MORE group. The traits with large differences in r_g estimate between the LESS and MORE groups are annotated. The colours of the dots indicate the trait categories defined as defined in LD Hub.

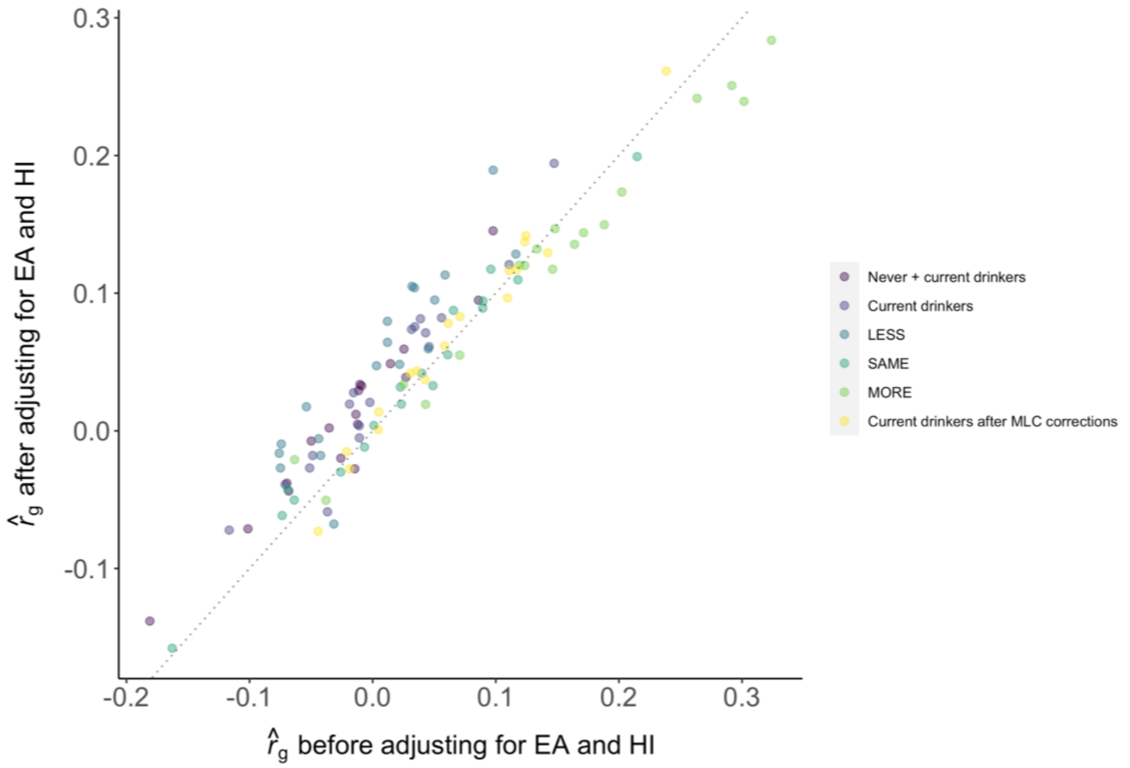

Supplementary Figure 12. Comparison of the estimates of genetic correlation between AC and 18 common diseases before and after adjusting for socio-economic status (SES). The x-axis and y-axis indicate the r_g estimates between AC and common diseases before and after adjusting two SES traits, educational attainment (EA) and household income (HI). The color of the circle indicates six different scenarios of AC GWAS. LESS, SAME, and MORE represent current drinkers whose AC levels were reduced, maintained the same, or increased, respectively, compared to 10 years ago. The r_g estimates are largely consistent before and after adjusting for EA and HI (Pearson's correlation r = 0.951). The Pearson's correlations in the subgroups are 0.967, 0.966, 0.895, 0.991, 0.987, 0.988, respectively, from the top to the bottom as shown in the legend.

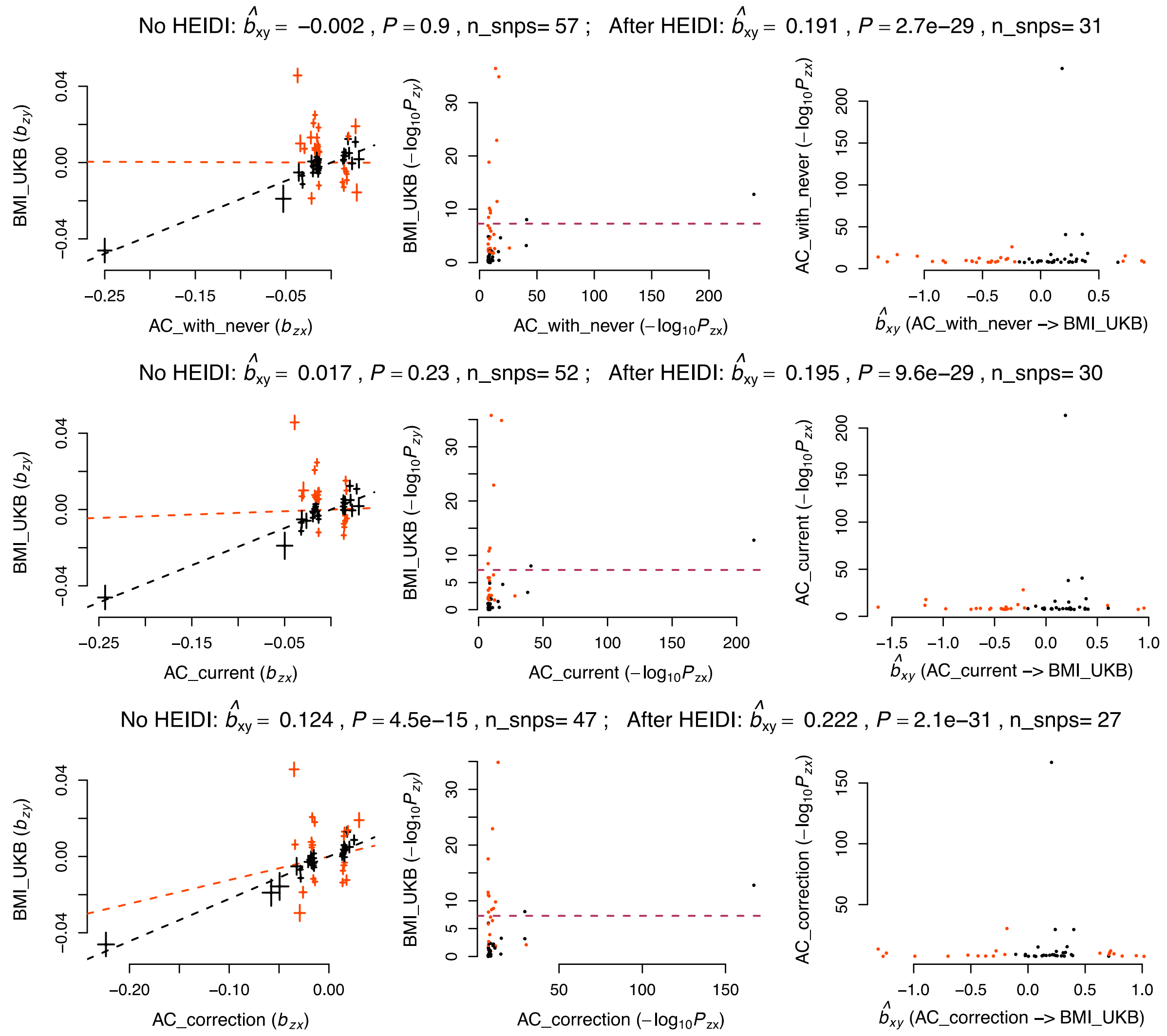

Supplementary Figure 13. GSMR diagnostic analysis of the causal association between AC and BMI in the UKB. The genetic instruments, which were detected by the HEIDI-outlier test as pleiotropic outliers, are highlighted in red. The three panels on the left show the estimated effects of the genetic instruments (index SNPs) of AC (x-axis) against those for BMI (y-axis). The error bars indicate the standard errors of the SNP effect estimates. The slope of the red and black dashed line indicates $\hat{b}_{xy}$ (GSMR estimate of the causal effect of AC on BMI) before and after the HEIDI-outlier filtering, respectively. The panels in the middle shows a plot of -log$_{10}$(P_{zx} or P_{zy}) for the effect of an index SNPs on the exposure (x-axis) against that for the outcome (y-axis). The panels on the right show the $\hat{b}_{xy}$ estimated using each index SNP (x-axis) against -log$_{10}$(P_{zx}) for the SNP effect on the exposure (y-axis). "AC_with_never": AC of current and never drinkers; "AC_current": AC of current drinkers; "AC_correction": AC after the MLC corrections. The b_{xy} is estimated based on a two-step least squares (2SLS) approach, and P-value indicates the significant level of the $\hat{b}_{xy}$ in GSMR analysis (two-sided test).

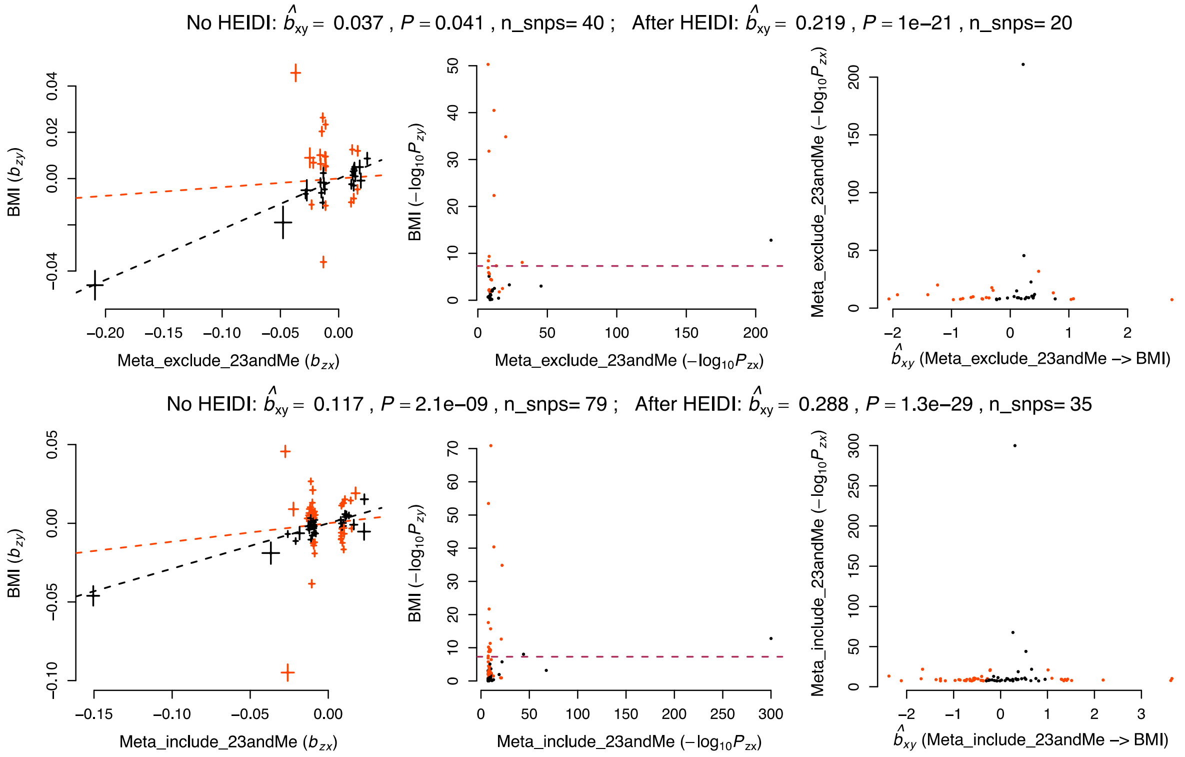

Supplementary Figure 14. GSMR diagnostic analysis of the causal association between AC and BMI using the UKB and GSCAN data. The genetic instruments, which were detected by the HEIDI-outlier test as pleiotropic outliers, are highlighted in red. The two panels on the left show the estimated effects of the genetic instruments (index SNPs) of AC (x-axis) against those for BMI (y-axis). The error bars indicate the standard errors of the SNP effect estimates. The slope of the red and black dashed line indicates $\hat{b}_{xy}$ (GSMR estimate of the causal effect of AC on BMI) before and after the HEIDI-outlier filtering, respectively. The panels in the middle shows a plot of -log10(P_{zx} or P_{zy}) for the effect of index SNPs on the exposure (x-axis) against that for the outcome (y-axis). The panels on the right show the $\hat{b}_{xy}$ estimated using each index SNP (x-axis) against -log10(P_{zx}) for the SNP effect on the exposure (y-axis). "Meta_exclude_23andMe" and "Meta_include_23andMe" represent the GSCAN data[4] of AC excluding and including 23andMe cohort, respectively. The b_{xy} is estimated based on a two-step least squares (2SLS) approach, and P-value indicates the significant level of the $\hat{b}_{xy}$ in GSMR analysis (two-sided test).

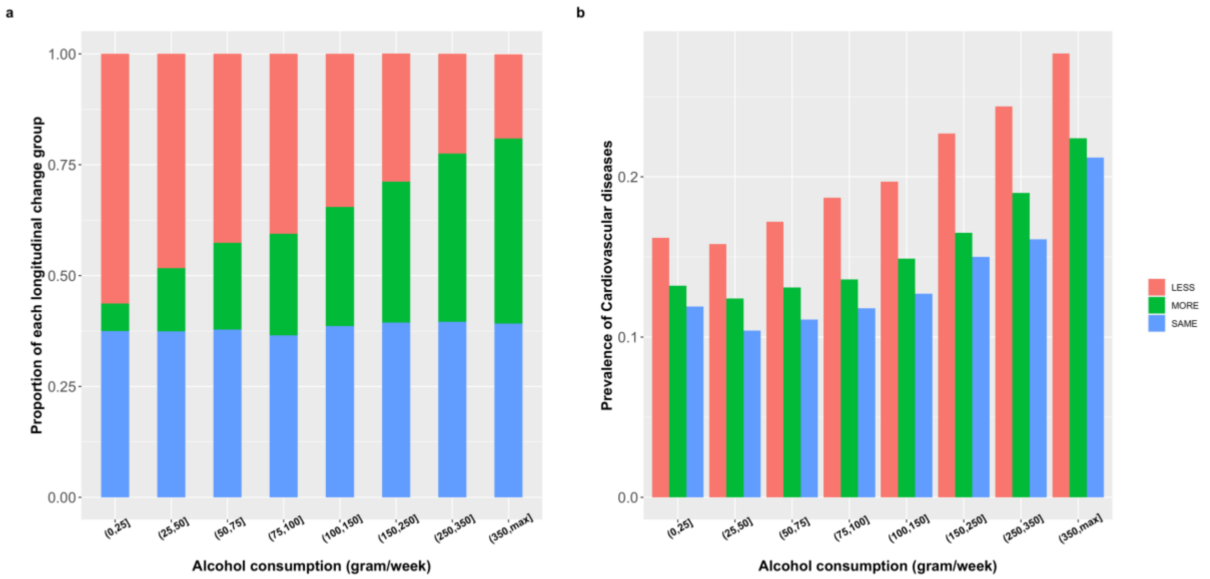

Supplementary Figure 15. Proportion of longitudinal change patterns and CVD prevalence in different AC level groups. (a) The x-axis shows eight AC level groups (measured by grams/week) as defined by the criteria in Wood et al.[3]. The y-axis shows the proportion of each longitudinal change group. (b) The y-axis denotes the prevalence of cardiovascular diseases. This x-axis is the same as in panel (a).

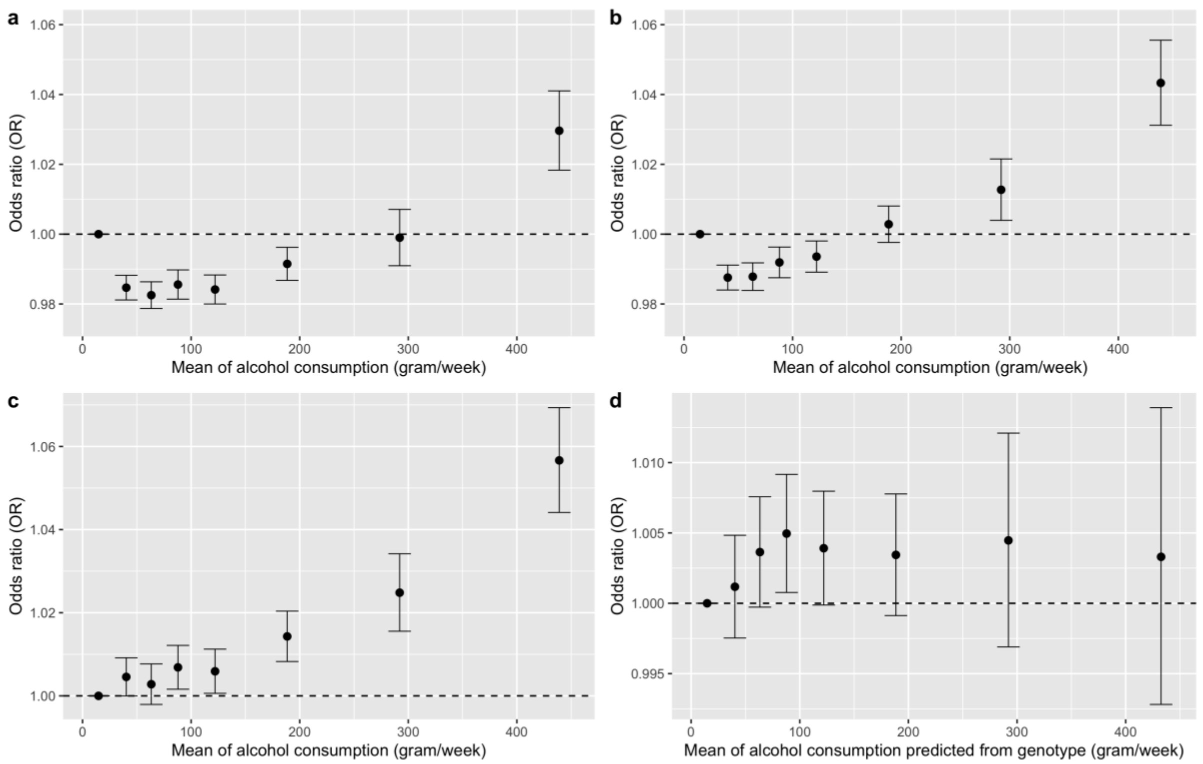

Supplementary Figure 16. The relationship between alcohol consumption and cardiovascular disease risk. The x-axes in panels a-c denote the mean alcohol consumption (gram/week) in each intake level group. The y-axes in all the panels denote the cardiovascular disease risk, measured by odds ratio (OR), against the reference group (0 < intake level ≤ 25 grams/week). (a) The regression was performed in all current drinkers (n = 356,138). (b) The individuals likely to underreport AC or reduce intake due to illness or doctor's advice were removed, and the logistic regression was adjusted for the longitudinal changes (n = 347,356). (c) Individuals from the LESS group were removed from the reference group (n = 319,320). (d) The x-axis denotes the genetically predicted alcohol consumption (n = 347,329). Each dot indicates the OR estimated against the reference group, and the error bars in all the panels indicate the 95% confidence intervals of the estimates.

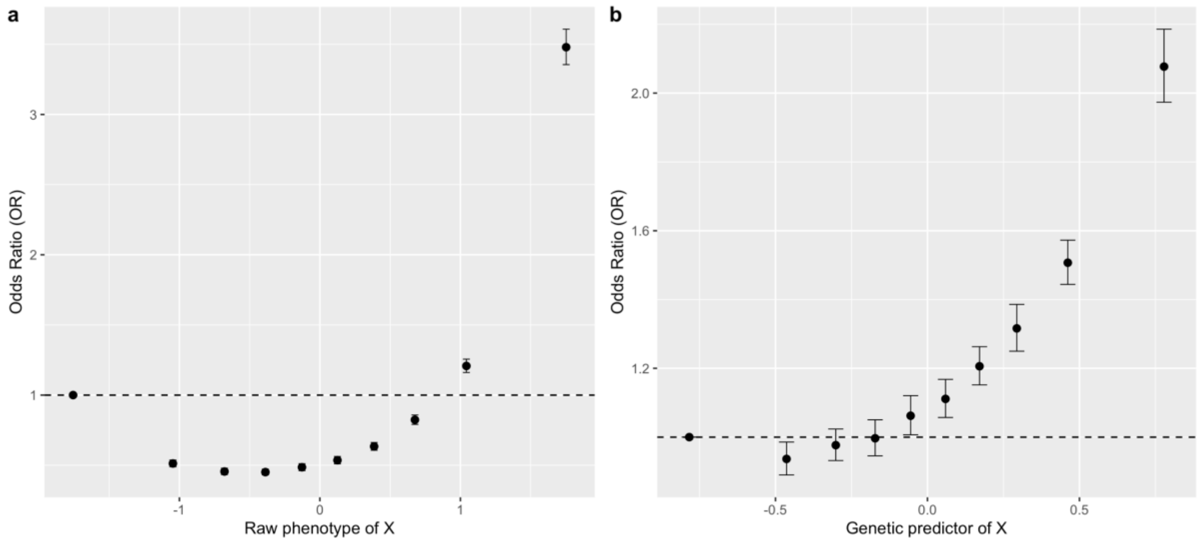

Supplementary Figure 17. J curve relationship between the genetic predictor of a trait X and the raw phenotype of a trait Y if the true relationship between X and Y is J-shaped. This figure demonstrates that if we predict X from genotypes, it is expected to have a J curve relationship with the raw phenotype of Y if the true relationship is a J curve. The total sample size used in the simulation $n = 50{,}000$. (a) The x-axis represents the simulated phenotypic value of X divided into 10 deciles. The y-axis represents the disease risk, as measured by odds ratio (OR) of each decile against the reference group (the first decile of X. (b) The x-axis represents the genetic predictor of X divided into 10 deciles. The y-axis represents the OR of each decile against the first decile. Each dot indicates the OR estimated against the reference group, and the error bars in all the panels indicate the 95% confidence intervals of the estimates.

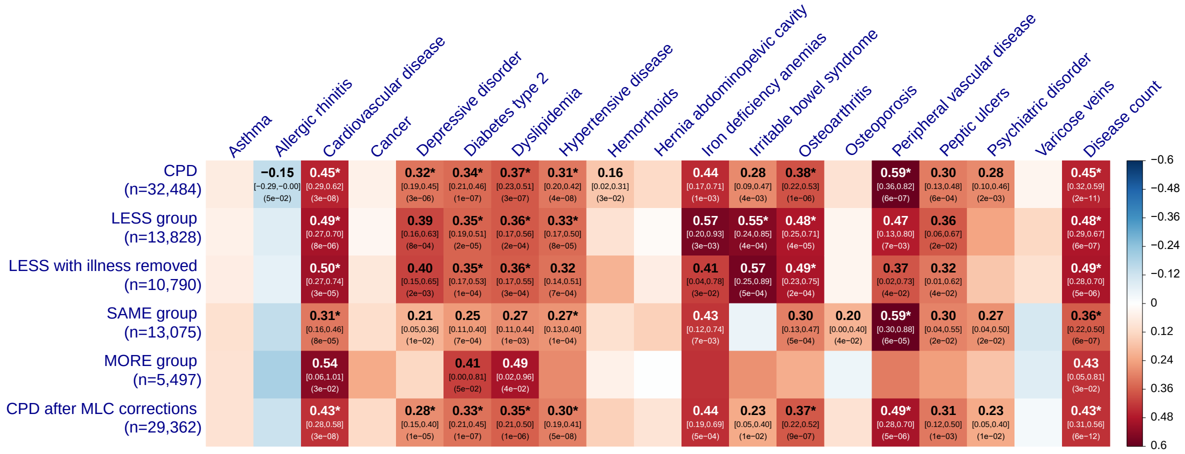

Supplementary Figure 18. Estimates of genetic correlation between cigarettes per day and common diseases in the UKB. The rows denote 6 GWAS summary data sets for cigarettes per day (CPD). The columns are 18 common diseases as well as disease count. The nominally significant estimates (P-value < 0.05) are labelled with the $\hat{r}_g$ [95% confidence interval] (P-value), and the significant estimates after multiple corrections (P-value < 0.05/114) are labelled with an additional asterisk. The genetic correlation is estimated from cross-trait LD score regression. The P-value shown in the block is the original P-value for $\hat{r}_g$ (two-sided χ^2test). CPD represents the CPD in all current smokers; LESS, SAME, and MORE groups represent the CPD within the group who reduced, maintained the same, or increased the amount of smoking, respectively, compared to 10 years ago. "LESS with illness removed" represents the CPD in the LESS group excluding individuals who reduced smoking due to illness or doctor’s advice. "CPD after MLC corrections" represents the CPD after the MLC corrections.

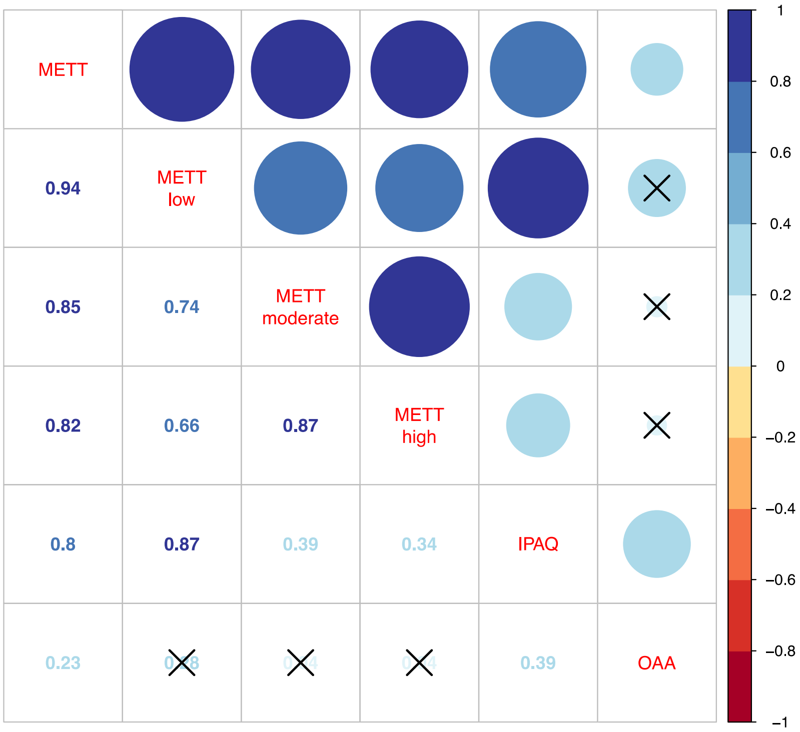

Supplementary Figure 19. Estimates of genetic correlation between physical activity traits. The value in each cell below the diagonal denotes the r_g estimate from a bivariate LDSC analysis. The circle in each cell above the diagonal shows the r_g estimate visually: larger circle size and darker color indicate higher r_g estimate. METT: Metabolic Equivalent Task in Total. IPAQ: International Physical Activity Questionnaire, short form. METT_low/moderate/high: METT in each of the three IPAQ categories. OAA: overall acceleration average measured by wrist-worn accelerometers. The estimates with P-value > 0.05 are annotated with a cross.

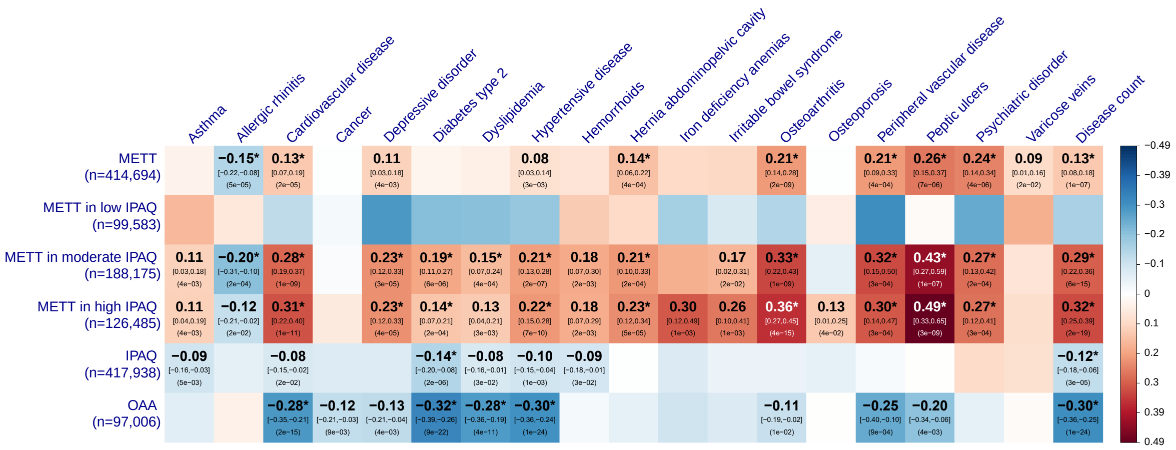

Supplementary Figure 20. Estimates of genetic correlation between physical activity traits and 18 common diseases. METT: Summed MET minutes per week for all activities. METT_low/moderate/high IPAQ: METT in each of the three IPAQ categories. IPAQ: International Physical Activity Questionnaire, short form. OAA: overall acceleration average. The columns are 18 common diseases along with disease count. The nominally significant estimates (P-value < 0.05) are labelled with the $\hat{r}_g$ [95% confidence interval] (P-value), and the significant estimates after multiple corrections (P-value < 0.05/114) are labelled with an additional asterisk. The genetic correlation is estimated from cross-trait LD score regression. The P-value shown in the block is the original P-value for $\hat{r}_g$ (two-sided χ^2 test).

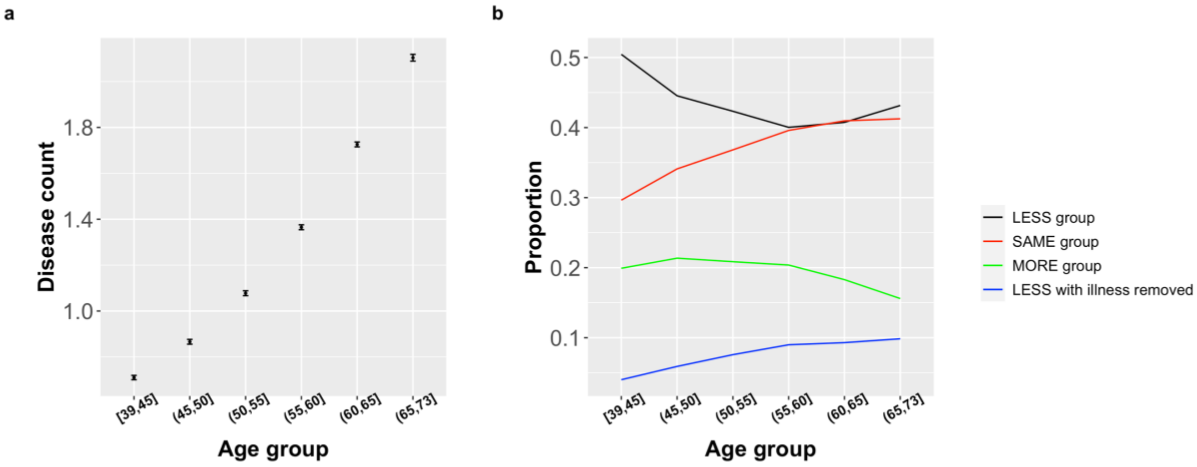

Supplementary Figure 21. Disease count and ascertainment are age dependent. The x-axis indicates 6 different age groups. (a) The y-axis indicates the average disease count in each age group. Each dot indicate the mean disease count in each age group, with the error bars indicating 95% confidence intervals. (b) The y-axis indicates the proportion of each longitudinal change group. The four groups are annotated by different colours. "LESS with illness removed" represents individuals who reduced drinking because of illness or doctor’s advice, compared to 10 years ago.

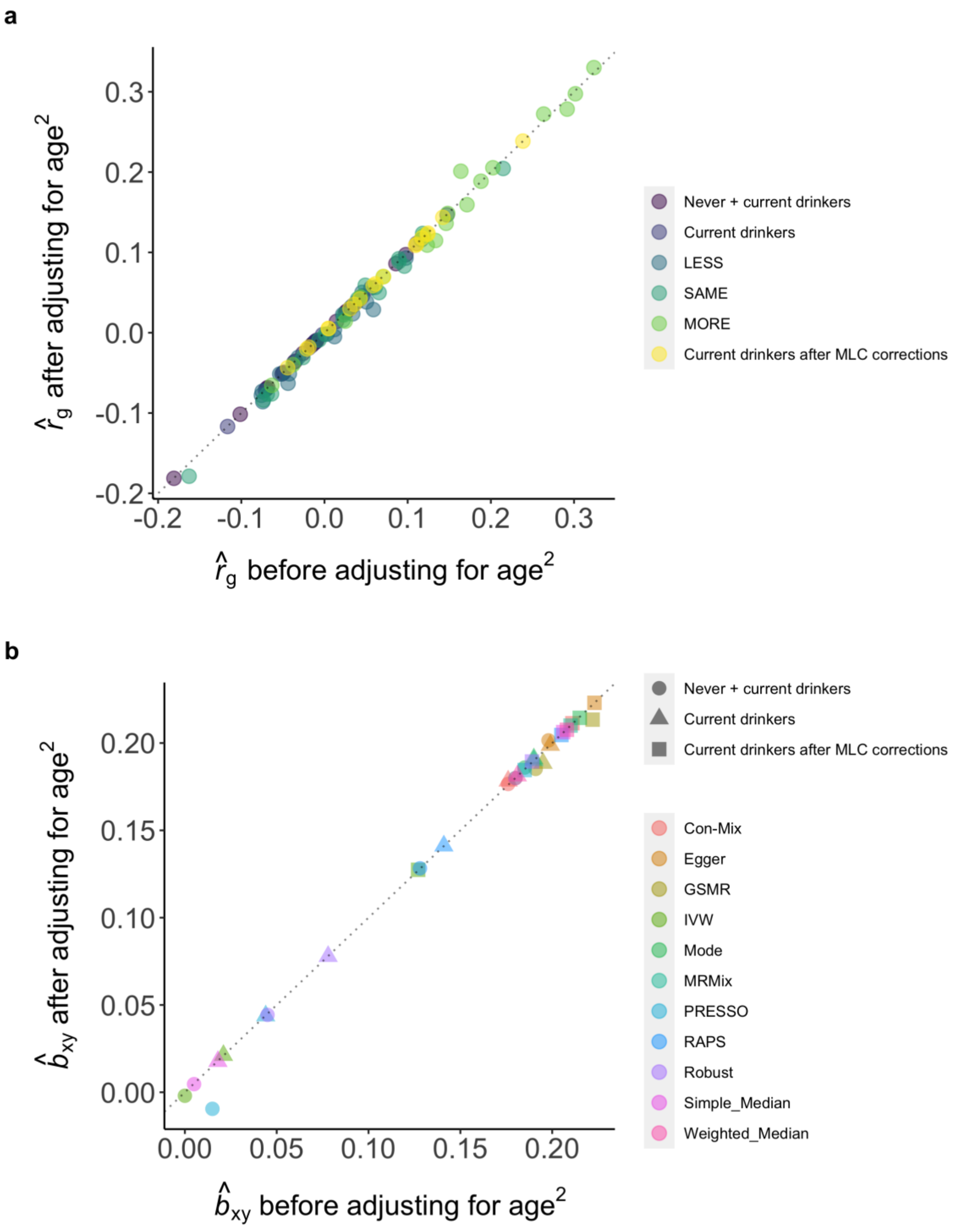

Supplementary Figure 22. Comparison of the estimates of genetic correlation or causal association between AC and BMI before and after adjusting for age^2. Panels (a) and (b) shows the results for genetic correlation and causal effect, respectively. The grey dashed line is the diagonal line. In panel (a), the color of the dot indicates different GWAS scenarios. In the panel (b), the shape of the point indicates different GWAS scenarios, and the color indicates different MR methods.

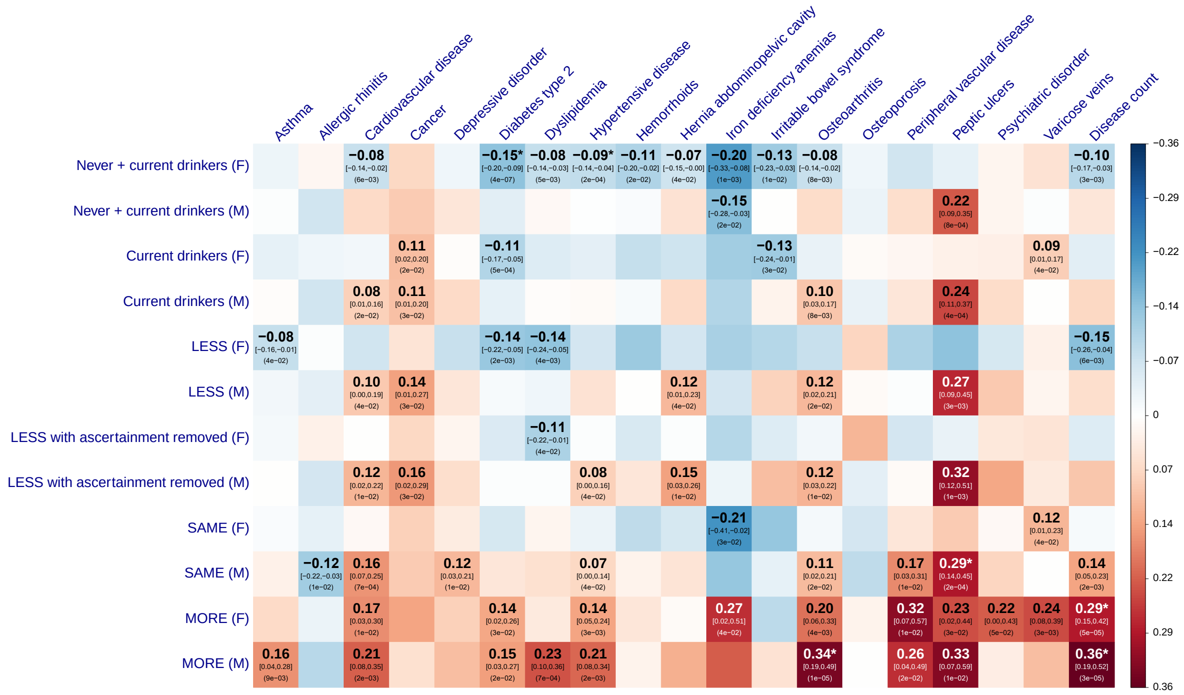

Supplementary Figure 23. Estimates of genetic correlation between AC and 18 common diseases in males and females separately under different scenarios. The rows denote 12 GWAS summary data sets for AC with the sex group labelled in the bracket ("F" for females and "M" for males). The columns are 18 common diseases along with the disease count. The nominally significant effects (P < 0.05) are labelled with r_g [95% confidence interval] (P-value), and the significant effects passing multiple testing correction (P < 0.05/228) are labelled with an additional asterisk. The genetic correlation is estimated from cross-trait LD score regression. The P-value shown in the block is the original P-value for $\hat{r}_g$ (two-sided χ^2test). LESS, SAME, and MORE represent current drinkers whose AC levels were reduced, maintained the same, and increased, respectively, compared to 10 years ago. "LESS with ascertainment removed" represents the LESS group excluding the participants who reduced their AC intake level due to illness or doctor's advice.

Supplementary References

1. Klatsky, A.L., Gunderson, E.P., Kipp, H., Udaltsova, N. & Friedman, G.D. Higher prevalence of systemic hypertension among moderate alcohol drinkers: an exploration of the role of underreporting. *Journal of Studies on Alcohol* **67**, 421-8 (2006).
2. Qi, T. *et al.* Identifying gene targets for brain-related traits using transcriptomic and methylomic data from blood. *Nat Commun* **9**, 2282 (2018).
3. Wood, A.M. *et al.* Risk thresholds for alcohol consumption: combined analysis of individual-participant data for 599 912 current drinkers in 83 prospective studies. *Lancet* **391**, 1513-1523 (2018).
4. Liu, M. *et al.* Association studies of up to 1.2 million individuals yield new insights into the genetic etiology of tobacco and alcohol use. *Nat Genet* **51**, 237-244 (2019).
