# Supplementary material for: Genome-wide analyses of behavioural traits are subject to bias by misreports and longitudinal changes
Source: Nat Commun. 2021 Jan 7;12:6450. doi: 10.1038/s41467-020-20237-6 (PMC7804181; doi:10.1038/s41467-020-20237-6)
Supplement: Supplementary file 3 — Reporting Summary [file 41467_2020_20237_MOESM3_ESM.pdf]

nature research

Corresponding author(s): Jian Yang

Last updated by author(s): Nov 7, 2020

Reporting Summary

Nature Research wishes to improve the reproducibility of the work that we publish. This form provides structure for consistency and transparency in reporting. For further information on Nature Research policies, see Authors & Referees and the Editorial Policy Checklist .

Statistics

For all statistical analyses, confirm that the following items are present in the figure legend, table legend, main text, or Methods section.

n/a	Confirmed	
☐	☒	The exact sample size (*n*) for each experimental group/condition, given as a discrete number and unit of measurement
☐	☒	A statement on whether measurements were taken from distinct samples or whether the same sample was measured repeatedly
☐	☒	The statistical test(s) used AND whether they are one- or two-sided<br>*Only common tests should be described solely by name; describe more complex techniques in the Methods section.*
☐	☒	A description of all covariates tested
☐	☒	A description of any assumptions or corrections, such as tests of normality and adjustment for multiple comparisons
☐	☒	A full description of the statistical parameters including central tendency (e.g. means) or other basic estimates (e.g. regression coefficient) AND variation (e.g. standard deviation) or associated estimates of uncertainty (e.g. confidence intervals)
☐	☒	For null hypothesis testing, the test statistic (e.g. *F*, *t*, *r*) with confidence intervals, effect sizes, degrees of freedom and *P* value noted<br>*Give P values as exact values whenever suitable.*
☒	☐	For Bayesian analysis, information on the choice of priors and Markov chain Monte Carlo settings
☒	☐	For hierarchical and complex designs, identification of the appropriate level for tests and full reporting of outcomes
☐	☒	Estimates of effect sizes (e.g. Cohen's *d*, Pearson's *r*), indicating how they were calculated

Our web collection on statistics for biologists contains articles on many of the points above.

Software and code

Policy information about availability of computer code

Data collection	We performed the LD score regression analyses online using the summary statistics curated in LD Hub to estimate the genetic correlations between alcohol drinking and 234 complex traits. We performed the PheWAS analysis using the Release 1 GWAS data (v20190117).
Data analysis	We used PLINK v1.90beta for quality controls of the genotyped or imputed SNP data, BOLT-LMM, v2.2 for GWAS analyses, and LDSC v1.0.0 for the estimation of SNP-based heritability and genetic correlation. We performed MR analyses using the R package 'MendelianRandomization' v0.4.1, MR-PRESSO v1.0, MRMix v0.1.0, RAPS v0.2, and GSMR implemented in GCTA v1.91.8 beta1. Data visualization was performed in R studio v1.2.1335 with R version 3.6.0. The simulation study was performed in R version 3.2.0.

For manuscripts utilizing custom algorithms or software that are central to the research but not yet described in published literature, software must be made available to editors/reviewers. We strongly encourage code deposition in a community repository (e.g. GitHub). See the Nature Research guidelines for submitting code & software for further information.

Data

Policy information about availability of data

All manuscripts must include a data availability statement. This statement should provide the following information, where applicable:

- Accession codes, unique identifiers, or web links for publicly available datasets
- A list of figures that have associated raw data
- A description of any restrictions on data availability

The GWAS summary data generated in this study are available at http://fastgwa.info/share/mlc-paper/. The individual-level genotype and phenotype data from the UKB are open to all bona fide researchers upon application (https://www.ukbiobank.ac.uk/principles-of-access/). The GWAS summary statistics in the PheWAS database can be downloaded at https://atlas.ctglab.nl/. The full download links of GWAS summary statistics in the LD Hub can be found in the Lookup Center after login in at http://ldsc.broadinstitute.org/. The 1000 Genome Project data can be downloaded at https://www.internationalgenome.org/data/.

Field-specific reporting

Please select the one below that is the best fit for your research. If you are not sure, read the appropriate sections before making your selection.

- [x] Life sciences
- [ ] Behavioural & social sciences
- [ ] Ecological, evolutionary & environmental sciences

For a reference copy of the document with all sections, see nature.com/documents/nr-reporting-summary-flat.pdf

Life sciences study design

All studies must disclose on these points even when the disclosure is negative.

Sample size	The main analysis was performed on 455,607 individuals of European ancestry in the UK Biobank (UKB). This sample size was decided by the maximum number of individuals of European ancestry within UKB to maximize the statistical power.
Data exclusions	In the UK Biobank data, we excluded the individuals who are not of European ancestry and removed SNPs with a minor allele count < 5, Hardy-Weinberg equilibrium test P-value < 1E-6, missing genotype rate > 5%, or imputation info score < 0.3.
Replication	We did not conduct any replication study because the self-reported longitudinal change information is unique in the UK Biobank. However, we did try to use the summary statistics of alcohol consumption from 23andMe to run the genetic correlation analysis and compared the results with that from the UK Biobank.
Randomization	Randomization is not relevant to this study. The participants of UKB are entirely voluntary.
Blinding	We did not have blinding design since this study did not include any clinical trials

Reporting for specific materials, systems and methods

We require information from authors about some types of materials, experimental systems and methods used in many studies. Here, indicate whether each material, system or method listed is relevant to your study. If you are not sure if a list item applies to your research, read the appropriate section before selecting a response.

Materials & experimental systems

n/a	Involved in the study	
x		Antibodies
x		Eukaryotic cell lines
x		Palaeontology
x		Animals and other organisms
	x	Human research participants
x		Clinical data

Methods

n/a	Involved in the study	
x		ChIP-seq
x		Flow cytometry
x		MRI-based neuroimaging

Human research participants

Policy information about studies involving human research participants

Population characteristics	The human research participants in the UK Biobank have an age range between 37 and 73 across the UK. Out of 455,607 participants of European ancestry, there are 247,188 females and 208,419 males. The genotype data were obtained using a customized Affymetrix Axiom array. The raw genotype data were imputed into the Human Reference Consortium (HRC) panel by the UKB team. We only included a subset of individuals of European ancestry in the analysis.
Recruitment	This study makes use of data from the UK Biobank (project ID: 12505). The UK Biobank is a national and international health resource, with ~500,000 volunteer participants providing genotypic and phenotypic information. Participants were recruited between 2006 and 2010, and attended one of the 22 assessment centers in the UK.
Ethics oversight	The UK Biobank has approval from the North West Multi-centre Research Ethics Committee (MREC), which covers the UK. It also sought approval in England and Wales from the Patient Information Advisory Group (PIAG) for gaining access to information that would allow it to invite people to participate. PIAG has since been replaced by the National Information Governance Board for Health & Social Care (NIGB). In Scotland, UK Biobank has approval from the Community Health Index Advisory Group (CHIAG).

Note that full information on the approval of the study protocol must also be provided in the manuscript.
