# Supplementary material for: Genome-wide analyses of behavioural traits are subject to bias by misreports and longitudinal changes
Source: Nat Commun. 2021 Jan 7;12:6450. doi: 10.1038/s41467-020-20237-6 (PMC7804181; doi:10.1038/s41467-020-20237-6)
Supplement: Supplementary file 4 — Description of Additional Supplementary Files [file 41467_2020_20237_MOESM4_ESM.pdf]

Description of Additional Supplementary Files

File Name: Supplementary Data 1

Description: Descriptive characteristics for 18 common diseases from UK Biobank; Disease prevalence in different drinking status groups; Disease prevalence for all current drinkers in different AC response and longitudinal change groups; Disease prevalence stratified by the reason of reducing intake in the LESS group.

File Name: Supplementary Data 2

Description: GWAS results for alcohol consumption before and after the MLC corrections in UK Biobank.

File Name: Supplementary Data 3

Description: PheWAS results of 30 down-sampling replicates and 16 "false positive" loci and 10 "false negative" loci.

File Name: Supplementary Data 4

Description: Estimates of genetic correlation between alcohol consumption and common diseases.

File Name: Supplementary Data 5

Description: Estimates of SNP-based heritability for AC related traits.

File Name: Supplementary Data 6

Description: Estimates of genetic correlation between pairwise AC related traits.

File Name: Supplementary Data 7

Description: Estimates of genetic correlation between alcohol consumption and 234 complex traits using data from LD-Hub.

File Name: Supplementary Data 8

Description: Estimates of genetic correlation between AC and BMI.

File Name: Supplementary Data 9

Description: Estimates of genetic correlation between AC and socio-economic traits.

File Name: Supplementary Data 10

Description: Estimates of causal effect of alcohol consumption on BMI using different MR methods.

File Name: Supplementary Data 11

Description: Estimates of causal effect of BMI on alcohol consumption by GSMR.

File Name: Supplementary Data 12

Description: Descriptive statistics of current smokers in different longitudinal change groups and descriptive statistics of CPD stratified by reasons for reducing smoking in the LESS group

File Name: Supplementary Data 13

Description: Estimates of genetic correlation between smoking intensity and common diseases.

File Name: Supplementary Data 14

Description: Disease prevalence in different smoking status groups; Disease prevalence of current cigarette smokers in different longitudinal change groups; Disease prevalence stratified by reason of reducing smoking in the LESS group.

File Name: Supplementary Data 15

Description: Estimates of genetic correlation between physical activity and common diseases.
