# Supplementary material for: Genome-wide analyses of behavioural traits are subject to bias by misreports and longitudinal changes
Source: Nat Commun. 2021 Jan 7;12:6450. doi: 10.1038/s41467-020-20237-6 (PMC7804181; doi:10.1038/s41467-020-20237-6)
Supplement: Supplementary file 2 — Peer Review [file 41467_2020_20237_MOESM2_ESM.pdf]

Reviewer #1 (Remarks to the Author):

Xue et al. present an interesting study on the alcohol consumption conundrum, where small amounts of AC have previously been reported to have beneficial health effects. This is however an incredibly hard question to answer for a number of reasons, namely because of misreports and longitudinal changes in AC, as well as various ascertainment biases in sample selection. The authors then propose and adjust the AC GWAS analysis for MLC, and find that this adjustment markedly changes the genetic architecture of AC. They also demonstrate using simulations how MLC can have such a large impact on AC. Generally, they find that the negative impact of AC on health is strengthened after the adjustment. However, given the many different possible sources of confounding, it is hard to draw any strong conclusions. The authors are well aware of these caveats, and list many of them in the discussion. In fact, I found few if any strong conclusive statements about the impact of AC on health, which in my opinion is good because it is still possible that the analysis is biased. It of course also means that the paper is not sensational and the discussion of the results may even seem dull. However, I think one cannot answer this important question in a decisive manner (at least not using UKB), and I found their analytical approach to the problem to be outstanding. In summary, the authors make a strong case for reducing AC, and I have no major comments.

Comments:
- Regarding the MLC adjustment, is there a worry that you're adjusting for heritable traits? Aschard et al., AJHG 2015 showed that adjusting for heritable traits can induce spurious signals. It seems to me that the simulation setup is aimed at addressing this concern, but as I understand it, it also assumes that D and Y do not share any common genetic variants, beyond what is induced through the effect of Y on D. Basically, I think it's hard to argue that the adjustment cannot induce a bias in some scenarios. In fact in line 163 you say "estimate became non-significant after the MLC corrections (Figure 3), likely because MLC are associated with EA", suggesting that you are aware of this bias. Maybe you could address this in text, or even in simulations (and maybe even prove me wrong).

- I think the introduction would be improved if you could spend a couple of sentences in the last paragraph on summarizing how you actually adjust capture and adjust for MLC (more details). I think it could help the flow of the paper.

- What is the take-home message of figure 1? Why is it interesting that 44.9% of associated phenotypes were metabolic/cardiovascular traits?

- In the MR comparison, why not compare with LCV (O'Connor and Price, NG 2018) as well?

- How about adjusting age^2 as a covariate as well. The reason is that disease liability is not linear in age, and AC probably isn't either.

- In Suppl. Figure 13. If we predict AC from genotype, would we really expect a J curve if the true relationship is a J curve? This is at least not obvious to me.

- In the physical activity analysis, you found a positive genetic correlation between all three measures, but given those I am surprised to see the change in directions of genetic correlations in supl. fig. 18. Please elaborate on this.

- Even though the Pirastu et al. (bioRxiv 2020; https://doi.org/10.1101/2020.03.22.001453) isn't out yet, given the relation with this paper, I believe it's worth mentioning and discussing. How would the bias described in that work impact these results?

Minor comments:
- Line 70. Why citation 24. This is a conclusion based on the data being analyzed in this paper. Maybe you can say something like "... problematic, as has previously been reported (citation)."

- Supplementary figure 4 had some font issues in caption in pdf. Also, what is r_b ?

- Several suppl. Figures partially obstructed captions in the pdf.

Reviewer #2 (Remarks to the Author):

This is an interesting paper and an important topic to get a better hold on given that the field depends a lot on self-reported behavioral outcomes.

I have some questions and suggestions that might help to improve the impact of the work.
I will report these as they came up to me while reading the paper from the beginning to the end. The list does not reflect any importance of one over the other and also includes minor remarks

1. P 2-3. Sometimes percentages are used, sometimes absolute numbers. For clarity and interpretation please report both percentages and absolute numbers in the text and the tables

2. Line 116 'than what is expected from a loss of sample' Pleas be more concrete and report the numbers to enable the interpretation and value of 'Significantly larger'
(I found it in the methods section, but prefer more concrete numbers at this stage in the paper)

3. Line 130 I do not think this should be table 1?

4. line 131 Supplementary table numbers are not correct.

5. L164. The authors refer to EA in this line and I think that this topic needs way more attention. Bias and is reporting is not EA independent and probably there is no linear relationship. Change in AC is also not EA independent and I expect that higher educated people change more than lower educated people. Furthermore, AC is also linked to student settings which makes changing AC for higher educated people easier (it is relatively easy to drink less than while at university). The effects of EA should be taken into account in several of the analyses and studied as a factor that influences the misreport and longitudinal change

6. It would be helpful to get more info on disease. In the methods there is a reference to Zhu et al., and info can be found on the UKB website, but given the importance of variable, it seems reasonable to give a more detailed description in the manuscript.

7. I, furthermore, wonder if the analyses could be more informative if they go beyond 'simple' disease count? Some diseases might be more related to misreporting and longitudinal change than others? More details are provided in supple table 1, and within these 18 diseases some are expected, as mentioned above, to be more or less related to misreporting. Furthermore, there is also a different association between different diseases and lifestyle factors such as AC, exercise, and smoking (e.g. somatic versus psychiatrics diseases).

8. Curious to get some more explanation of possible interpretation for the finding that rg between AC and common disease varied by LESS, SAME, and MORE group, while this effect is absent for CPD. So MLC seems to have a different effect in AC than in smoking. Focus in the discussion section is mainly on AC, while I think that the differences in effects of MLC for smoking and exercise deserves more discussion.

9. The section on physical activity (L240- 259) is not very clear. Why are these different measures used? It is well known that there is not much overlap between reported physical activity and accelerometer assessments. Why is one considered to be more biased than the other? Object activity assessment in no way error free. Please include what is known from the literature about the reliability of these measures and what could explain the pattern of rg with the diseases.

10. To my opinion the discussion section lacks a proper discussion on the possible reasons for MLC. One of the factors could for example be that someone's view on alcohol has changed over time. What was considered not much 10 years back might be considered a lot when you are 10 years older. Furthermore, the general acceptance of alcohol use has also changed substantially over the past years, in parallel with the increased consumption (and social acceptance) of alcohol-free beverages. This also has an effect on socially desirable answering patterns that have different effects on retrospective reporting than on real-time reporting.

10. A surprising observation was the lower BMI in the participants that reported an increase in AC (table 1). Is this a significant difference (probably not)? Could this be an effect that is driven by EA?

11. The possible important role of EA is also reflected in table 2 where I observe (and correct me if I am wrong) that. Reducing due to health precaution is related to higher EA (and lowest BMI, so maybe even more than EA including also SES), indicating that the reason for change is not independent of EA while misreporting is also not independent of EA.

REVIEWER COMMENTS

We thank the two reviewers for their constructive comments, which have helped us to improve this manuscript. We have responded to all the reviewers' comments point-by-point below (in blue) and have highlighted all the relevant changes (in yellow) in the revised manuscript and supplementary materials.

Reviewer #1 (Remarks to the Author):

Xue et al. present an interesting study on the alcohol consumption conundrum, where small amounts of AC have previously been reported to have beneficial health effects. This is however an incredibly hard question to answer for a number of reasons, namely because of misreports and longitudinal changes in AC, as well as various ascertainment biases in sample selection. The authors then propose and adjust the AC GWAS analysis for MLC, and find that this adjustment markedly changes the genetic architecture of AC. They also demonstrate using simulations how MLC can have such a large impact on AC. Generally, they find that the negative impact of AC on health is strengthened after the adjustment. However, given the many different possible sources of confounding, it is hard to draw any strong conclusions. The authors are well aware of these caveats, and list many of them in the discussion. In fact, I found few if any strong conclusive statements about the impact of AC on health, which in my opinion is good because it is still possible that the analysis is biased. It of course also means that the paper is not sensational and the discussion of the results may even seem dull. However, I think one cannot answer this important question in a decisive manner (at least not using UKB), and I found their analytical approach to the problem to be outstanding. In summary, the authors make a strong case for reducing AC, and I have no major comments.

Re: We thank the reviewer for the summary and for the positive remarks. We agree that our paper certainly does not end the debate regarding the effect of alcohol consumption (AC) on health outcomes but provides plausible explanations to some of the inconsistent observations from previous studies and an empirical approach to attenuate biases due to MLC in the UKB data. We acknowledge that even after the proposed MLC corrections, the estimates are not necessarily unbiased. We therefore caution the interpretation of the observed associations of behavioural phenotypes with health and advocate more specifically designed questionnaires for behavioural phenotypes that are subject to MLC.

Comments:

1) Regarding the MLC adjustment, is there a worry that you're adjusting for heritable traits? Aschard et al., AJHG 2015 showed that adjusting for heritable traits can induce spurious signals. It seems to

me that the simulation setup is aimed at addressing this concern, but as I understand it, it also assumes that D and Y do not share any common genetic variants, beyond what is induced through the effect of Y on D. Basically, I think it's hard to argue that the adjustment cannot induce a bias in some scenarios. In fact in line 163 you say "estimate became non-significant after the MLC corrections (Figure 3), likely because MLC are associated with EA", suggesting that you are aware of this bias. Maybe you could address this in text, or even in simulations (and maybe even prove me wrong).

Re: In our MLC corrections, we did not adjust for any heritable traits. In brief, we excluded participants with unreliable self-reported records, ran GWAS in three longitudinal groups separately (with sex, age and PCs fitted as covariates) and then meta-analysed the summary statistics from the separate GWAS analyses. We have clarified this in the revised manuscript (lines #55-61 and lines #425-438).

In the simulation, we also did not adjust D for Y (or Y for D). The aim of the simulation is not to address the issue raised by Aschard et al., but to mimic a disease ascertainment on a modifiable exposure, i.e., a high value of D tends to lead to a reduction in Y. Such ascertainment induces a spurious negative phenotypic or genetic correlation between D and Y even if they do not share any genetic variants, and the magnitude of the correlation is proportional to the strength of the ascertainment (**Supplementary Figure 5A**).

In line 163 (lines #172-174 in the revised version), we show that MLC could also bias the genetic correlation estimates between AC and socio-economic status (SES) and conclude that misreporting and longitude changes are not independent of SES. This conclusion is supported by the summary results presented in **Table 2** that the participants who reduced AC because of "health precaution" have a higher mean educational attainment level than that in all the other categories. As noted by the reviewer, we are fully aware of collider bias. Therefore, in the additional analysis to adjust AC for SES traits (lines #183-191 and **Supplementary Figure 12**), we chose to use mtCOJO, a method that has been shown to be robust to collider bias (Zhu et al. 2018. *Nat Communications*).

2) I think the introduction would be improved if you could spend a couple of sentences in the last paragraph on summarizing how you actually adjust capture and adjust for MLC (more details). I think it could help the flow of the paper.

Re: We thank the reviewer for this suggestion. We have added the following sentences toward the end of the introduction section (lines #55-61).
"We then propose a correction procedure to mitigate the MLC biases. Take AC as an example. We identify and remove the participants whose self-reported AC is inconsistent with their intake

frequency, medical records or online follow-ups and the participants who reduced their AC intake because of illness or doctor's advice during the past 10 years. Then, we stratify the participants into three longitudinal change groups (drink “less”, “the same” or “more” compared to 10 years ago) and run a GWAS analysis in each group separately followed by a meta-analysis. We also elaborate on why some of the previous studies might suffer from MLC biases."

3) What is the take-home message of figure 1? Why is it interesting that 44.9% of associated phenotypes were metabolic/cardiovascular traits?

Re: **Figure 1** shows associations of the AC-associated variants, which became non-significant because of the MLC corrections, with the common traits and diseases for which summary data from large-scale GWASs are available in the public domain (https://atlas.ctglab.nl/PheWAS). The main purpose of showing this figure is to visualise the traits and diseases that show an enrichment of association with the AC signals removed by the MLC corrections. We observed an enrichment of association for metabolic/cardiovascular diseases and then tested the statistical significance of the enrichment by a resampling technique (lines #120-125). We have added a few sentences in the legend of **Figure 1** for clarification (page #20).

4) In the MR comparison, why not compare with LCV (O’Connor and Price, NG 2018) as well?

Re: We thank the reviewer for this comment, which has inspired us to include more MR methods (including MR-Egger, MRMix, Con-Mix, Mode, RAPS and Robust) in the comparison (**Figure 4** on page #23).

We did not include LCV before because strictly speaking LCV is not an MR method although it can be used for causal inference. The estimate from LCV, called genetic causality proportion (GCP), quantifies the proportion of genetic component of the exposure that is causal for the outcome. GCP ranges from 0 (no partial genetic causality) to 1 (full genetic causality) which has a very different interpretation from the causal effect estimated by the MR methods.

5) How about adjusting age^2 as a covariate as well. The reason is that disease liability is not linear in age, and AC probably isn't either.

Re: We have re-run the genetic correlation and MR analyses with age^2 fitted as an additional covariate for AC (**Supplementary Figure 22** and lines #318-321). The results remained nearly identical (Pearson's correlation *r* between the results with and without adjusting for age^2 was 0.997 for the genetic correlation estimates and 0.999 for the MR estimates).

6) In Suppl. Figure 13. If we predict AC from genotype, would we really expect a J curve if the true relationship is a J curve? This is at least not obvious to me.

Re: We have tested this by an additional simulation in the revised manuscript (**Supplementary Note 3** and **Supplementary Figure 17**). We simulated a J-shaped relationship between the exposure and the outcome (**Supplementary Note 3**). We then used genome-wide significant variants to generate a polygenic risk score (PRS) for the exposure and estimated the effect of the PRS on the outcome in different quantiles of the exposure. The relationship was still a J-shaped curve, supporting the hypothesis that if the true relationship between AC and health is J-shaped, we would expect to see a J-shaped relationship between AC PRS and health.

7) In the physical activity analysis, you found a positive genetic correlation between all three measures, but given those I am surprised to see the change in directions of genetic correlations in supl. fig. 18. Please elaborate on this.

Re: We included three commonly used physical activity (PA) measurements in this study, i.e., METT, IPAQ, and OAA. These three measurements are expected to be positively genetically correlated with each other because all of them are PA indicators. However, this does not necessarily mean that their correlations with diseases should be in a consistent direction because one measurement could suffer more from disease ascertainment biases than another giving rise to a change of the sign of the genetic correlation estimate for some PA-disease pairs but not for the others. For example, METT is subject to misreporting while OAA, a device-based measurement, is very unlikely to be biased by misreporting. Although the phenotypic and genetic correlation between self-reported and device-measured PA are not expected to be unity, if there is no MLC bias, the correlation should have been higher. We have commented on this and expanded the discussion for the PA traits in the revised manuscript (lines #282-288).

8) Even though the Pirastu et al. (bioRxiv 2020; https://doi.org/10.1101/2020.03.22.001453) isn't out yet, given the relation with this paper, I believe it's worth mentioning and discussing. How would the bias described in that work impact these results?

Re: We thank the reviewer for this comment. We agree that gender difference is likely to be one of the sources of the MLC bias. For example, if we look at the gender ratio in each of the longitudinal change groups (LESS, SAME and MORE), there are more males who reduced AC (84266/175761 = 47.9%) than females (68588/180756 = 37.9%). The male/female ratio are 1.22, 0.95, and 0.59 in the LESS, SAME and MORE groups, respectively. In our AC GWAS analysis, we removed mean and

variance differences between the gender groups for AC by standardizing AC in females and males separately. However, if some of the trait-associated alleles are more frequent in one gender group (as pointed out in Pirastu et al. bioRxiv 2020) and there are genotype-sex interaction effects, the difference in longitudinal change between females and males will cause a bias in AC GWAS (as part of the MLC bias). Such sex-differential longitudinal change bias can be alleviated by the MLC corrections as demonstrated in our additional analysis (**Supplementary Figure 23**). We have commented on this issue in the revised manuscript (lines #361-369).

Minor comments:
9) Line 70. Why citation 24. This is a conclusion based on the data being analyzed in this paper. Maybe you can say something like "... problematic, as has previously been reported (citation)."

Re: We have revised the text as per the reviewer's suggestion (line #77).

10) Supplementary figure 4 had some font issues in caption in pdf. Also, what is r_b ?

Re: We have fixed the font issues.
The r_b is defined as Pearson's correlation between the effects of the genetic variants on Y and those on D accounting for errors in the estimated variant effects. We have clarified this in the revised manuscript (**Supplementary Note 2** and **Supplementary Figure 5**).

11) Several suppl. Figures partially obstructed captions in the pdf.

Re: Fixed.

Reviewer #2 (Remarks to the Author):

This is an interesting paper and an important topic to get a better hold on given that the field depends a lot on self-reported behavioral outcomes.

I have some questions and suggestions that might help to improve the impact of the work.
I will report these as they came up to me while reading the paper from the beginning to the end. The list does not reflect any importance of one over the other and also includes minor remarks

1. P 2-3. Sometimes percentages are used, sometimes absolute numbers. For clarity and interpretation please report both percentages and absolute numbers in the text and the tables

Re: We have reported both percentages and absolute numbers wherever appropriate in the main text and the tables.

2. Line 116 'than what is expected from a loss of sample' Please be more concrete and report the numbers to enable the interpretation and value of 'Significantly larger'
(I found it in the methods section, but prefer more concrete numbers at this stage in the paper)

Re: We have reported the actual numbers in the revised text (lines #120-125).
"We showed by a down-sampling analysis that the number of loci that became non-significant after the MLC corrections (16) was significantly larger than that expected from a loss of sample size (10.03, *s.e.* = 0.49), and 10 loci that became genome-wide significant after the MLC corrections were likely to be masked by MLC in the uncorrected GWAS (the expected number is 3.26, *s.e.* = 0.30, **Methods**, **Supplementary Tables 2-3**)."

3. Line 130 I do not think this should be table 1?

Re: We apologise for the typo. It should be **Figure 1** and **Supplementary Table 4**.

4. line 131 Supplementary table numbers are not correct.

Re: Apologies again for the typo. We have corrected the supplementary table numbers.
"Before the MLC corrections, we observed substantial differences between $\hat{r}_g$ (between AC and diseases) estimated using AC GWAS data from the LESS, SAME and MORE groups (**Figure 1** and **Supplementary Table 4**). We also estimated the SNP-based heritability (h^2_{SNP}) from different AC

GWAS data sets and r_g between the data sets (**Supplementary Tables 5-6**, and **Supplementary Figure 10**) and found that the $\hat{r}_g$ between AC in the LESS and MORE groups was significantly different from unity ($\hat{r}_g = 0.796$, standard error ($s.e.$) $= 0.074$)."

We thank the reviewer for picking up these typos. We have taken this opportunity to have careful proofreading of the revised manuscript to avoid mistakes.

5. L164. The authors refer to EA in this line and I think that this topic needs way more attention. Bias and is reporting is not EA independent and probably there is no linear relationship. Change in AC is also not EA independent and I expect that higher educated people change more than lower educated people. Furthermore, AC is also linked to student settings which makes changing AC for higher educated people easier (it is relatively easy to drink less than while at university). The effects of EA should be taken into account in several of the analyses and studied as a factor that influences the misreport and longitudinal change

Re: We agree with the reviewer that misreporting and longitudinal changes are not EA independent. In fact, we have observed in the UKB data that the participants who reduced AC because of "health precaution" have a higher mean EA level than that in all the other categories (**Table 2**). It is also likely that misreporting and longitudinal changes depend on other socio-economic traits such as household income (HI).

To test the effects of EA and HI on our results, we adjusted AC for EA and HI. To avoid collider bias due to adjusting for a heritable phenotype, we performed the adjustment using the mtCOJO approach which is more robust to collider bias than the conventional covariate adjustment approach (Zhu et al. 2018. *Nat Communications*). We found that before the MLC corrections, the genetic correlation (r_g) estimates between AC and 18 common diseases after further EA and HI adjustment were highly consistent with those before the adjustment (Pearson's correlation $r = 0.966$) (**Supplementary Figure 12**). The consistency was even higher after the MLC corrections ($r = 0.988$) (**Supplementary Figure 12**). These results suggest that biases in AC GWAS due to EA and HI are likely to be small and have largely been removed by the MLC corrections.

We have included these additional results and discussion in the revised manuscript (lines #183-191; **Supplementary Figure 12**).

6. It would be helpful to get more info on disease. In the methods there is a reference to Zhu et al., and info can be found on the UKB website, but given the importance of variable, it seems reasonable to give a more detailed description in the manuscript.

Re: We have added the ICD codes of the diseases and the numbers of cases and controls in **Supplementary Table 1A** and a few sentences in the main text to explain how we selected the diseases (lines #414-417).

7. I, furthermore, wonder if the analyses could be more informative if they go beyond 'simple' disease count? Some diseases might be more related to misreporting and longitudinal change than others? More details are provided in supple table 1, and within these 18 diseases some are expected, as mentioned above, to be more or less related to misreporting. Furthermore, there is also a different association between different diseases and lifestyle factors such as AC, exercise, and smoking (e.g. somatic versus psychiatrics diseases).

Re: We agree that some diseases, such as cardiovascular disease, type 2 diabetes, dyslipidemia, hypertensive disease, and iron deficiency anemias, seem to be more related to MLC for AC than others (**Figure 1**, **Figure 2** and **Supplementary Table 1**). We understand the comment "beyond a simple disease count" as a count that gives diseases different weights, which, however, is difficult to achieve. On one hand, deriving the weights from the results from the same data is biased because of the existing ascertainment of the estimation errors. An unbiased approach would be to obtain the weights from an independent data set with longitudinal records, which is currently infeasible because UKB is currently the only large data sets with all the MLC information available.

8. Curious to get some more explanation of possible interpretation for the finding that rg between AC and common disease varied by LESS, SAME, and MORE group, while this effect is absent for CPD. So MLC seems to have a different effect in AC than in smoking. Focus in the discussion section is mainly on AC, while I think that the differences in effects of MLC for smoking and exercise deserves more discussion.

Re: We had provided a plausible explanation of why we observed a difference in the MLC bias pattern between AC and CPD in the Results section of the previous manuscript (lines #234-240 previously). In the revised manuscript, we have expanded the discussion and moved the text to the Discussion section (lines #342-351).

The main reason for the difference is likely to be that the participants in the LESS group had a much higher mean disease count than those in both the SAME and MORE groups for AC (**Table 1**), indicating strong disease ascertainment, whereas such disease ascertainment was not apparent for CPD, e.g., the mean disease count in the LESS group is lower than that in the MORE group (**Supplementary Table 12**). More specifically, in the LESS group for CPD, the illness subgroup (i.e.,

participants reduced CPD because of illness) has a higher mean CPD level than the other subgroups (**Supplementary Table 12B**), whereas in the LESS group for AC, the illness subgroup has a lower mean AC level (7.33 units/week) than the other subgroups (8.63 units/week). We hypothesize that these differences are because the likelihood of whether people choose to stop or reduce smoking due to reasons such as illness is different from that for drinking, e.g., when affected by illness, people tend to quit rather than reduce smoking but tend to reduce rather than stop drinking. This hypothesis is supported by the observations in the UKB that ~77% of the ever smokers are former smokers (**Supplementary Table 14A**) while only ~3% of the ever drinkers are former drinkers (**Supplementary Table 1B**).

9. The section on physical activity (L240- 259) is not very clear. Why are these different measures used? It is well known that there is not much overlap between reported physical activity and accelerometer assessments. Why is one considered to be more biased than the other? Object activity assessment in no way error free. Please include what is known from the literature about the reliability of these measures and what could explain the pattern of rg with the diseases.

Re: We picked up these three measures because they are the most commonly used indicators for physical activities and available in the UK Biobank. We have clarified this in the text (line #265).

According to the literature, the genetic overlap between self-reported PA and accelerometer assessments is not high, consistent with our results. We agree that accelerometer assessments also suffer from certain types of biases, but according to the literature, device-measured PA show consistent inverse relationship with BMI, blood pressure, and adiposity, while self-reported PA show inconsistent genetic correlation patterns at different intensity levels (Doherty et al 2018. Nat Commun.; Klimentidis et al. 2018. Int. J. Obes.). Previous work also pointed out that self-reported records in elder cohorts could suffer more from recall bias due to a high proportion of cognitive impairment (Innerd et al. 2015. Age and Ageing). The questionnaire-based measurement showed varied reliability in different populations and sociodemographic groups (Craig et al. 2003. Medicine & Science in Sports & Exercise; Sabia et al. 2014. Am. J. Epidemiol).

Our r_g estimates with common diseases for IPAQ and OAA are largely consistent and altogether suggest a beneficial effect of PA on health, but the estimates of r_g for METT are remarkably different from and most of them are in the opposite direction with those from IPAQ and OAA. We do not have self-reported longitudinal change information as those for AC and CPD to investigate if the METT r_g results are biased by disease ascertainment, but we show indirectly that there is strong heterogeneity among the METT stratified by three IPAQ categories (**Supplementary Figure 20**). Together with the evidence from the literature, we conclude that IPAQ and OAA are better PA indicators than METT,

and the r_g results for METT are potentially to be biased by disease ascertainment. This conclusion is of course not definitive and needs to be confirmed in the future with more data. We have included this discussion in the revised manuscript (lines #282-288).

10. To my opinion the discussion section lacks a proper discussion on the possible reasons for MLC. One of the factors could for example be that someone's view on alcohol has changed over time. What was considered not much 10 years back might be considered a lot when you are 10 years older. Furthermore, the general acceptance of alcohol use has also changed substantially over the past years, in parallel with the increased consumption (and social acceptance) of alcohol-free beverages. This also has an effect on socially desirable answering patterns that have different effects on retrospective reporting than on real-time reporting.

Re: We have included a proper discussion on the possible reasons for MLC in the revised manuscript (lines #342-351).
"Second, there are many reasons for MLC. These reasons include the self-reported reasons such as illness, doctor's advice, health precaution and financial issues, and other reasons such as social desirability, major life changes (e.g., change of marital status and having a child), influences from family members or friends, religious experience, self-evaluation and legal problem (Matzger et al. *Addiction*. 2005; Polcin et al. *Contemp Drug Probl*. 2012). In the UKB survey, ~58% of the individuals with reduced alcohol intake reported that the reduction was due to "other reasons" or "do not know" in the survey (**Table 2**). Any of the reasons especially those related to disease and health precaution, if not accounted for, would lead to biases in GWAS and subsequent analyses. Also, since social acceptance is an important factor for the MLC reasons, the change of social acceptance over time might give rise to differences in MLC between real-time and retrospective reports."

11. A surprising observation was the lower BMI in the participants that reported an increase in AC (table 1). Is this a significant difference (probably not)? Could this be an effect that is driven by EA?

Re: We thank the reviewer for pointing this out. This observation may be better interpreted as that participants with higher BMI tend to reduce AC. The difference in BMI between the LESS and SAME groups (Welch-t = 67.9, $-\log_{10}(P)$ = 841.0) and the difference between the LESS and MORE groups (Welch-t = 64.0, $-\log_{10}(P)$ = 879.7) are highly statistically significant. Thus, the observation is in line with the result from our reverse GSMR analysis that BMI has a decreasing effect on AC (page #6) and one of our conclusions that participants with cardiometabolic diseases tend to reduce AC because these diseases are often associated with higher BMI (page #7). This observation is unlikely to be driven by EA because the differences remain highly significant (Welch-t = 77.3, $-\log_{10}(P)$ = 1288.9

between LESS and SAME and Welch-t = 79.5, -$\log_{10}(P)$ = 1344.8 between LESS and MORE) after adjusting BMI for EA. We have commented on this issue in the revised manuscript (lines #191-197).

12. The possible important role of EA is also reflected in table 2 where I observe (and correct me if I am wrong) that. Reducing due to health precaution is related to higher EA (and lowest BMI, so maybe even more than EA including also SES), indicating that the reason for change is not independent of EA while misreporting is also not independent of EA.

Re: As stated in our response to comment #5 from this reviewer, we agree that MLC is not independent of EA and SES. We have re-run the genetic correlation (r_g) analyses after adjusting AC for EA and SES. The results showed that without the MLC corrections, the r_g estimates between AC and 18 common diseases after further EA and HI adjustment were highly consistent with those before the adjustment (Pearson's correlation r = 0.966) (**Supplementary Figure 12**). The consistency was even higher after the MLC corrections (r = 0.988). These results suggest that biases in AC GWAS due to EA and HI are likely to be small and have largely been removed by the MLC corrections. We have included these additional results and discussion in the revised manuscript (lines #183-191).

Reviewer #1 (Remarks to the Author):

I would like to thank the authors for addressing all of my comments.

Reviewer #2 (Remarks to the Author):

The authors took into account my comments and suggestion in a sufficient way
